# Using gene topologies to partition sex chromosomes into regions with distinct histories

**DOI:** 10.1093/molbev/msag249

**Published:** 2026-10-16

**Authors:** Suzie Tallon, Lila Maladesky, Roula Jabbour-Zahab, Aline Muyle, Thomas Lenormand

**Affiliations:** CEFE, Univ Montpellier, CNRS, EPHE, IRD, Montpellier, France; CEFE, Univ Montpellier, CNRS, EPHE, IRD, Montpellier, France; CEFE, Univ Montpellier, CNRS, EPHE, IRD, Montpellier, France; ISEM, Univ Montpellier, CNRS, IRD, Montpellier, France; CEFE, Univ Montpellier, CNRS, EPHE, IRD, Montpellier, France

**Keywords:** change-point analysis, *Silene*, *Artemia*, XY, ZW, strata

## Abstract

Sex chromosomes often evolve through successive recombination arrests, forming evolutionary strata. Accurately describing these strata, their boundaries and history is crucial for studying sex chromosome evolution, but this can be very challenging, particularly in young sex chromosomes where X–Y or Z–W divergence is low or in highly rearranged sex chromosomes where strata are divided into pieces. Standard approaches to distinguish recently evolved strata involve combining signals from dS, Fst and gene-by-gene phylogenies, all of which can be noisy. Here, we propose a new method that uses the proportion of allele topologies observed on sex-linked genes between at least a pair of sister species, obtained from reconstructed X and Y sequences aligned to the same reference, along with an outgroup to infer ancestral alleles, with flexibility in the reconstruction pipeline. We implement a change-point analysis to objectively identify transitions in these proportions. Our method both delimitates strata and infers whether their evolutionary history is shared or convergent between species. We illustrate and test this method on *Silene* and *Artemia* species. Our new method is reliable and robust, globally confirming previous findings known for some of these species. The method is also sensitive, refining those previous findings and identifying regions that were not previously reported. We finally illustrate how this approach can inform scenarios that retrace the history of sex chromosomes in these species. Overall, this approach presents a tool that can be widely used and that complement existing methods to study sex chromosomes.

## Introduction

Across the tree of life, sex chromosomes have evolved multiple times from autosomes. Well-studied examples include an XY system in placental mammals, a ZW system in birds or lepidoptera, both XY and ZW in angiosperms, and UV systems in certain algae and mosses (Ashman et al. 2014). Their origin often involves the emergence of a sex-determining locus on an autosome, followed by recombination suppression between the proto-X and proto-Y (or Z and W), the subsequent degeneration of the non-recombining chromosome, and the evolution of dosage compensation. While these events have been broadly documented, the processes responsible for each of these steps, along with their temporal sequence and underlying causes, remain under active discussion (Abbott et al. 2017; Charlesworth 2021; Lenormand and Roze 2024; Saunders et al. 2024; Charlesworth et al. 2025). Recombination suppression can evolve in multiple steps and take many forms, ranging from cases where only small genomic segments are affected to systems where large chromosomal regions cease to recombine at once (Wright et al. 2016; Furman et al. 2020; Charlesworth 2021), and even to taxa where recombination is naturally reduced or absent, as in achiasmate species (Satomura et al. 2019). In some cases, reversals or turnover events have also been described, where a new, non degenerated Y (or W) chromosome arises from its homologous X (or Z) (Beukeboom and Perrin 2014; Vicoso 2019; Zhu et al. 2024). As a result, in chiasmate species, sex chromosomes are often structured into evolutionary strata, which are regions with different levels of X–Y divergence, that reflect the successive events of recombination suppression along the X. Alongside these strata, pseudo-autosomal regions (PAR), located at one or both ends of the sex chromosomes, are areas where recombination still occurs between the X and Y (or Z and W), maintaining homology.

Accurately describing sex chromosome strata is an essential step for studying sex chromosome evolution. Precise delimitation of strata is necessary to unpack the history of sex chromosomes. It is required to uncover when recombination suppression evolved, which rearrangements were involved, if any, their size, and whether they occurred convergently or ancestrally in sister species. With this information available, it becomes possible to investigate and test the theories aimed at explaining recombination suppression, degeneration or dosage compensation. For instance, one can investigate whether stratum size is related to the extent of degeneration, how rapidly Y chromosome degeneration occurs in gene-rich young sex chromosomes, whether dosage compensation evolves early on or not (Bachtrog 2008; Muyle et al. 2022; Charlesworth et al. 2025). Annotating strata is also necessary to investigate the role of sex chromosomes and their degenerated and dosage compensated strata in speciation, including in phenomena such as Haldane’s rule and the large X/Z effect (Lenormand and Roze 2025). All these tests can only be reliable if the age, size and gene content of strata have been well annotated.

However, reconstructing sex chromosome strata and their evolutionary history is not straightforward. A striking example comes from guppies (*Poecilia reticulata*) and related species. Independent studies based on cytological and genomic data proposed different scenarios: some inferred large, ancient non-recombining strata shared among species, while others suggested more recent, independent recombination suppression in each species (Wright et al. 2017; Bergero and Charlesworth 2019; Darolti et al. 2020). Re-analyses of the data (Kirkpatrick et al. 2022) highlighted both the difficulties of studying those genomic regions and the value of studying this in closely related species. One major difficulty is to distinguish between regions that completely ceased to recombine and those where recombination is very rare, as both could show similar patterns of X–Y divergence. Another difficulty is to distinguish strata that convergently occurred at similar time points in each species separately, and differentiate them from ancestral strata, as these two scenarios can show similar patterns of X–Y divergence. These challenges lie at the core of ongoing efforts to precisely delimit strata, infer their history, their chromosomal location, their age and whether they are ancestral or convergent when comparing sister species.

Strata are usually identified as sex-linked genomic regions showing particular patterns that differ quantitatively from those observed in neighbouring regions. Apart from obtaining clean phased haplotypes for the different chromosomes that may become more and more available (see e.g. Wei et al. 2026), sex-linkage can be assessed from crosses, or genetic association to the sex of individuals in populations (Muyle et al. 2017; Palmer et al. 2019; Saunders et al. 2024). Male-to-female read depth ratios (with stringent mapping on an X or Z reference) are often taken as a measure of Y/W divergence. Synonymous divergence (dS) between X and Y gene copies is often used as a way to date recombination suppression. Male-female Fst is a way to identify regions that have accumulated some divergence between the sexes, as would be expected on the non-recombining parts of sex chromosomes. Current studies use these metrics alone or in combination (for recent case studies, see e.g. Wang et al. 2012; Vicoso et al. 2013; Wright et al. 2017; Darolti et al. 2020; Peichel et al. 2020; Prentout et al. 2021; Elkrewi et al. 2022; Filatov 2022; Sacchi et al. 2024; Moraga et al. 2025). Identifying strata is often done by performing change-point analyses on X–Y dS (e.g. Akagi et al. 2025a, 2025b; Moraga et al. 2025; Carpentier et al. 2026), Fst (e.g. Wang et al. 2024; Carpentier et al. 2026) and/or read depth ratio (e.g. Sardell et al. 2021). These analyses are done using generic change-point packages available in R (e.g. Killick and Eckley 2014; Lindeløv 2020), or using workflows that use those packages such as EASYstrata (Rougemont et al. 2025). These methods are useful for detecting relatively old strata where substantial divergence has accumulated on the X and Y. However, they can struggle to identify young strata. In this case, the X and Y would remain very similar, resulting in divergence-based measures being close to zero. Moreover, these divergence-based measures tend to be noisy, making them often insufficient to provide a reliable signal leading to the difficulties mentioned above: distinguishing genuinely recombining regions from regions where recombination has only recently stopped, and distinguishing strata of similar age.

Once strata are identified, evolutionary scenarios are typically tested in a second step using phylogenetic information (e.g. Handley et al. 2004; Wright et al. 2014; Darolti et al. 2020; Okuno et al. 2021; Prentout et al. 2021; Sardell et al. 2021; Zhang et al. 2022; Tessarotto et al. 2025). Consensus trees on genomic regions or genes are compared across species to determine, for instance, whether X and Y alleles cluster by chromosomes—consistent with recombination suppression occurring in the ancestor, before speciation—or by species—consistent with convergent evolution. This approach is most often used post hoc to study the history of regions of the chromosomes that were first delimited using measures of divergence. In 2022, Kirkpatrick et al. (2022) refined this approach by using allele topologies at the level of single nucleotide bases, instead of entire genes. Using allele topologies takes advantage of all the existing genetic diversity in X and Y chromosomes, instead of averaging the phylogenetic signal at the gene scale, which can blur alternative evolutionary scenarios. Kirkpatrick et al. (2022) were able to confirm convergent recombination suppression in guppies using this method. While Kirkpatrick et al. (2022) used allele topologies to test for evolutionary scenarios, they did not use it for direct strata annotation. As such, the full potential of allele topologies as a strata annotation signal has not yet been fully exploited.

In this paper, we propose to use the proportion of allele topologies directly as the primary signal for identifying and delimiting strata. By “allele topologies,” we refer to the genealogical relationships between X and Y alleles, at the level of a single nucleotide, across a pair of sister species. As illustrated on Fig. 1, when alleles group X chromosomes from different species together separately from Y chromosomes (or vice versa), it indicates ancestral recombination suppression, prior to speciation. Hereafter we refer to this pattern as “grouping by chromosomes”. On the other hand, when X–Y alleles cluster by species, it indicates convergent recombination suppression that occurred separately in each species after speciation. Hereafter we refer to this pattern as “grouping by species”. Finally, more complex patterns exist, such as those expected after a sex chromosome turnover event (Fig. 1). Our method also makes use of the proportion of singletons, corresponding to cases where only one chromosome in one species differs from other alleles. Indeed, the proportion of singletons may also vary if branch lengths differ across genomic regions (Fig. 1). The proportion of topologies along the X chromosome therefore carries a quantitative signal that can be exploited to identify strata boundaries and infer their evolutionary origin. Importantly, this signal reflects relative branch lengths rather than absolute divergence. Ancestral strata (where X and Y sequences may be more divergent within than between species) and convergent strata (where X and Y sequences may be less divergent within than between species) can both be detected. This allows the method to be applied to strata of different ages, from ancient to recent.

**Figure 1:**
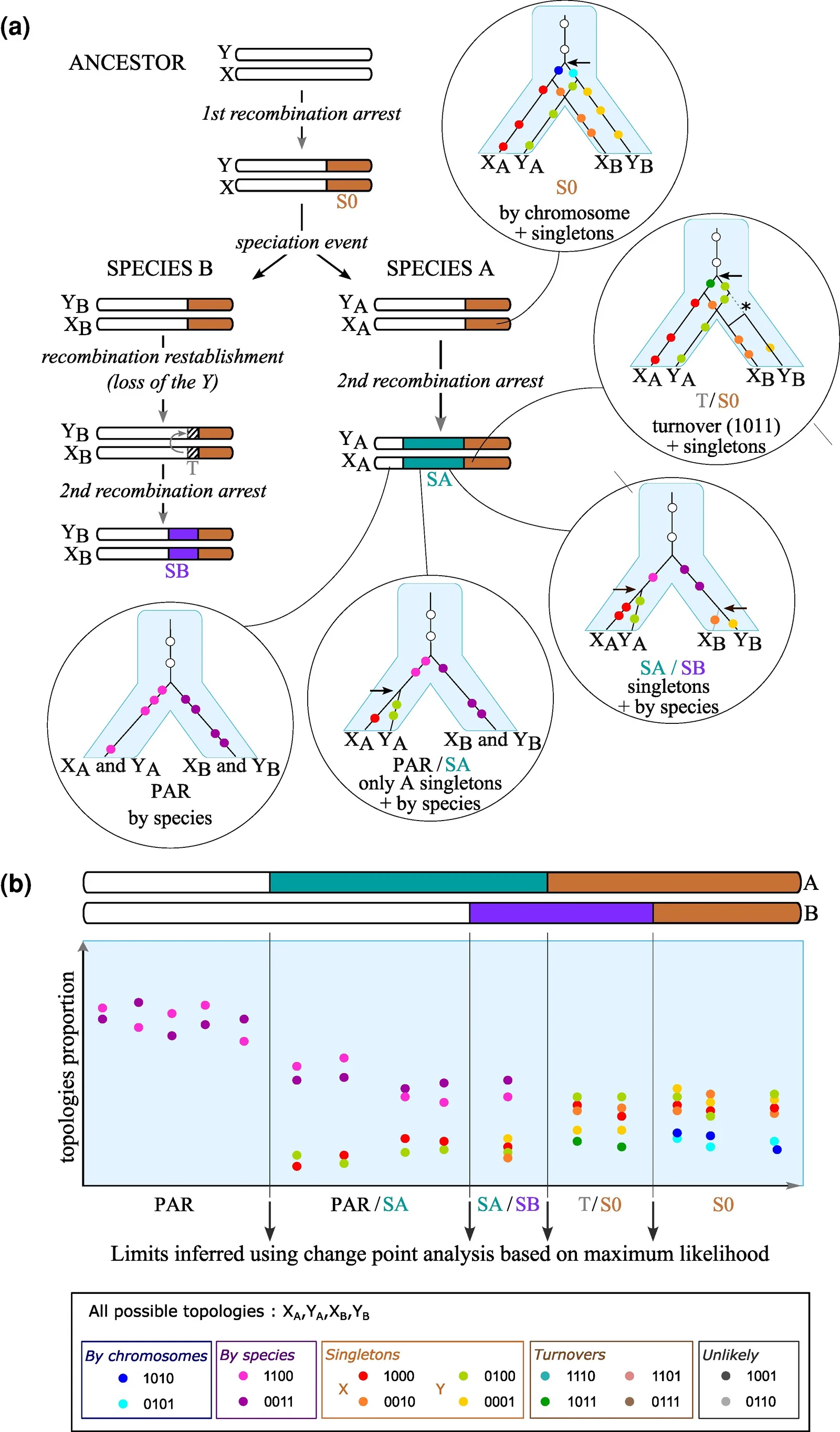
Schematic representation illustrating the principle of our new method using a hypothetical scenario. The scenario is shown in (A), where the different bubbles indicate the expected alleles in each region of the sex chromosomes. Arrows indicate recombination arrest and asterisks indicate turnover events. The blue enveloppe illustrates the speciation process. The figure shows a region where recombination was suppressed before speciation (S0 in brown) and two independently evolved strata in species A and B (in blue SA and purple SB, respectively). (B) shows the topological signature that should be observed in this hypothetical scenario when computing the proportion of each X–Y allele topology along the X chromosome. Topologies are coded as four digits, taking the value 0 for the ancestral allele inferred from an outgroup or 1 for the derived allele. The first two digits represent the X and Y alleles of the first species in the pair (species A), while the final two digits represent the X and Y alleles of the second species in the pair (species B).

This approach would be a useful tool to complement existing methods and would presents several advantages. First, our method uses allele topologies as a way to directly identify strata, rather than only as a post hoc tool to interpret the history of regions identified by other means. Second, it makes use of a rich signal—the variation in the proportions of many possible allelic topologies along the chromosome—which allows the detection of subtle changes, such as recent strata or strata of similar ages. In contrast, dS, Fst, or dXY-based methods rely on a single summary measure of divergence, which may lack the resolution to detect such fine-scale patterns. Third, it could be embedded in a likelihood-based statistical framework to objectively evaluate support for different models of strata. Fourth, it would also provide direct information about the historical scenarios that could have produced these strata: whether they are ancestral or independently evolved after speciation, and whether turnover events are plausible.

To illustrate the potential of this approach, we applied it to two independent systems with different modes of sex determination (XY and ZW). For a comparison, we also examined patterns of synonymous divergence (dS) for sex-linked genes in all species. The first system is the *Melandrium* section of the *Silene* genus, which includes five Eurasian perennial dioecious species (*Silene latifolia*, *Silene dioica*, *Silene diclinis*, *Silene heuffelii*, and *Silene marizii*). Their XY sex chromosomes are relatively young: the origin of sex chromosomes is estimated at ∼11 My (Krasovec et al. 2018), by estimating a mutation rate), though other studies suggest that the closest hermaphrodite species diverged only ∼5 My ago, implying a more recent origin (Rautenberg et al. 2010; Jenkins and Keller 2011; Rautenberg et al. 2012). The *Melandrium* section we investigated is estimated to have started diverging ∼3 My ago by those same studies, but finer-scale divergence time within the section remains unresolved. This system is also particularly well suited for our approach because a robust species phylogeny is available (Muyle et al. 2021), which provides a firm baseline for interpreting allele topologies. The second system is the brine shrimp genus *Artemia*, represented here by two Asian species, *Artemia sinica* and *Artemia urmiana*, whose divergence time is still debated, with estimates varying widely; however, Sainz-Escudero et al. (2021) suggest ∼6.6 My as the most likely date. *Artemia* possesses ZW sex chromosomes generally considered old, but this is also debated, as it remains unclear how many species in this clade share them. Elkrewi et al. (2022) showed that the ZW sex chromosome of *A. sinica* and *A. urmiana* is also ancestral in *Artemia franciscana*, whose divergence time is likewise uncertain but estimated by Sainz-Escudero et al. (2021) at ∼8.42 My. Despite this great uncertainty about the precise age of the sex chromosomes, several studies suggest the presence of younger strata as well (Elkrewi et al. 2022).

Importantly, evolutionary strata have already been described in at least one species in both *Silene* and *Artemia*, which allows us to compare our results with previous studies. In *S. latifolia*, three strata were identified by Moraga et al. (2025), with the third thought to result from a translocation from the X onto the Y within the oldest stratum. Additionally, Filatov (2022) reported a fourth, small stratum of 16 genes, which appears not to be shared with *S. dioica* or *S. diclinis*. In *S. diclinis*, a neo-Y chromosome has also been formed through a reciprocal translocation with chromosome 10 (Howell et al. 2009). In the brine shrimp *A. sinica*, two strata have been described by Elkrewi et al. (2022). The first, older stratum is shared with *A. franciscana*, a more distantly related species. The second is a more recent stratum that appears to have evolved independently.

We illustrate with these two case studies how the method can be used broadly. The two systems offer indeed a diversity of situations (plant and animal, XY and ZW, recent and ancestral strata) typical of the existing range of sex chromosome strata diversity in eukaryotes.

## Material and methods

In this paper, we propose a new method for identifying sex chromosome strata using variation in the proportions of allele topologies along a reference X chromosome. To apply this method, a critical preliminary step is to recover reliable X and Y (or Z and W) sequences, typically through SNP calling. In this study, we used a pipeline to reconstruct these sequences from RNA-seq and laboratory cross data using the SEX-DETector program (Muyle et al. 2016). However, this is not the only way to obtain X and Y sequences. Other data types, such as genomic, RAD-seq, or pool-seq, can also be used. Similarly, laboratory crosses are not mandatory. For example, SDpop, which relies on population data from males and females (Käfer et al. 2021), is another possible approach. The essential requirement is to have X and Y sequences for at least one pair of sister species and an outgroup, which are then aligned to a reference X, no matter how these sequences were obtained. Aligning SNPs to a high-quality reference is critical because our method relies on gene order (spatial information) to accurately delimit strata and reconstruct the events that led to recombination suppression along the sex chromosomes. While it is possible to align to both X and Y references, the X chromosome is more commonly used because high-quality X reference genomes are more widely available and Y chromosomes are notoriously difficult to assemble, as documented in (Tomaszkiewicz et al. 2017; Carey et al. 2022). Secondary rearrangements (e.g. inversions occurring after recombination suppression) may occur on both X and Y chromosomes. Our method will only detect secondary rearrangements on the chromosome used as a reference for SNP alignment. Aligning to the X is also a better option considering that the non-recombining part of the Y chromosome is likely to accumulate more of these secondary rearrangements. By aligning to an X reference, there is therefore a better chance of analyzing a simpler pattern, closer to the primary steps of recombination suppression.

The detailed pipeline used to process the data is summarized in online supplementary material, Fig. S1. Steps that can be performed starting from aligned X and Y alleles obtained through another pipeline are highlighted in purple. Many processing steps were performed using custom bash and R scripts (highlighted in bold in online supplementary material, Fig. S1), which are available on GitHub (https://github.com/suzie-tallon/topology_based_sex_chromosome_partition), using the packages tidyverse (Wickham et al. 2019), patchwork (Pedersen 2017), zoo (Zeileis and Grothendieck 2005), and reshape2 (Wickham 2007).

### Sequence data

For the two case studies *Silene* and *Artemia*, we used laboratory crosses, for each species, to identify consensus nucleotide sequences for the sex chromosomes (X and Y chromosomes in *Silene* ; Z and W chromosomes in *Artemia*). For each cross, we obtained RNA-seq data for one male (the father), one female (the mother), 4-5 of their male offspring and 4-5 of their female offspring.

For *Artemia*, we used laboratory strains originating from two natural populations: *A. sinica* from Xiechi Lake (Northern China) and *A. urmiana* from Urmia Lake (Iran). The cysts were provided by the *Artemia* Reference Center with the reference ARC1206 and ARC1230, respectively. Hatching and rearing conditions of individuals were as described in (Boyer et al. 2021). RNA was extracted from parents whole body and five of their adult offspring of each sex using a single-step RNA isolation protocol with NucleoZol (Macherey-Nagel, Düren, Germany), following the manufacturer’s instructions. Sequencing was performed by Genowiz on an Illumina NovaSeq platform, generating 2×150 bp paired-end reads. Some individuals were sequenced in two independent runs, and the resulting datasets were merged for downstream analyses. Sequencing data are available in the NCBI under project PRJNA1530745.

For *Silene*, RNA-seq data from crosses were obtained from previously published studies. For *S. latifolia*, maternal RNA-seq data are available in the GEO database (series GSE35563; Muyle et al. 2012), while sequences from the father and four offspring of each sex are archived in ENA under project PRJEB14171 (Muyle et al. 2016). For the other *Silene* species, sequencing data for the parents and five offspring of each sex are available in the NCBI under the project accession SUB13729399 (Prentout et al. 2021). In all these studies, RNA was extracted from floral buds at stages B1 to B2 (4 mm, early reproductive organ development) after calyx removal, and sequenced using comparable Illumina paired-end 150 bp protocols. *S. latifolia* individuals were sequenced once, whereas individuals from the other species were sequenced in three independent runs and merged for downstream analyses.

### Data cleaning, mapping, and genotyping

RNA-seq reads were quality-checked with FastQC v0.12.1 (Andrews 2010) and MultiQC v1.19 (Ewels et al. 2016). *Silene* reads were trimmed with Trim Galore v0.6.10 (Krueger et al. 2023), discarding reads shorter than 50 bp. *Artemia* reads were trimmed using Cutadapt v3.5 (Martin 2011). While low-quality bases are usually found at read ends, sudden drops in quality sometimes occurred mid-sequence. In these cases, trimming was performed in two steps: first, cutting reads at a fixed length (100 to 120 bp depending on the individual), and second, filtering by quality with a second Trim Galore run. Contamination was checked with FastQ Screen v0.15.3 (Wingett and Andrews 2018). One male offspring of the *A. urmiana* cross was contaminated by human DNA that we removed using kraken2 v2.1.5 (Wood et al. 2019). Reads with high ribosomal RNA content were removed using Ribodetector v0.3.1 (Deng et al. 2022). All filtering parameters are provided in online supplementary material, Table S1.

RNA-seq reads were mapped to the genome reference of either *S. latifolia* without the Y (Moraga et al. 2025) or ZZ male *Artemia sinica* (Elkrewi et al. 2022) using a more recent annotation than the one published with the original paper, which is available on their GitHub repository (Elkrewi et al. 2026). Using the X or Z chromosome is standard to annotate strata, as it allows to work with the ancestral sequence ordering at the time when Y and W rearrangements evolved. In some species X rearrangements have been documented, but, most of the time, the Y is rearranged at a considerably higher rate than the X. Alignments were performed with STAR v2.7.11a (Dobin et al. 2013), using the –quantMode TranscriptomeSAM option to retain only transcriptomic alignments. For multimapping reads, STAR was instructed to randomly assign a primary site (–outMultimapperOrder Random). Secondary alignments were removed with Samtools v1.19 using -F 0x100, and unmapped reads were filtered with -F 4. Multiple sequencing runs per individual were merged at this stage.

Genotyping was carried out with Reads2snp v2.1 (Gayral et al. 2013) using parameters tailored for sex-linked loci: -aeb to account for allelic expression bias (this is important since Y alleles are often under-expressed, making it harder to genotype the Y allele in RNA-seq data), -bqt 20 to retain bases with quality ≥20, -rqt 0 to avoid discarding Y reads with lower mapping quality, -min 3 to require at least three reads supporting each genotype call, and -par 0 to keep putative paralogs with paraclean, as filtering them might remove true X/Y SNPs.

### Sex-linked allele inference and curation of false positives

We inferred sex-linked SNPs using the SEX-DETector pipeline (Muyle et al. 2016), run with default parameters, which detects sex-linked alleles and assigns them to the X or Y chromosome in *Silene* and to the Z or W chromosome in *Artemia*. For example, in *Silene*, if the father is AT, the mother AA, all four male offspring AT, and all four female offspring AA, then the T allele is most likely Y-linked and the A allele is most likely X-linked. Conversely, in *Artemia*, if the mother is AT, the father AA, all female offspring AT, and all male offspring AA, then the T allele is inferred to be W-linked and the A allele Z-linked. This approach also identifies which regions of the sex chromosomes are recombining (PAR) and which are non-recombining, since sex-linked genes are expected only in the latter.

Few isolated false positives can nevertheless be observed within the PAR because of sampling variance. In addition, since only a few meiosis and crossing-overs (CO) are sampled in a family cross, some genes may appear to be sex-linked in the cross, when in fact they belong to the PAR. This would occur notably in the region between the true PAR limit and the position of the closest CO sampled in the cross. To filter out such cases in *Silene*, we used additional RNA-seq data from females collected in natural populations: if an allele inferred as Y-linked was also present in any of these females, the SNP was reclassified as autosomal and removed using a custom R script. Population RNA-seq data, extracted and sequenced using protocols similar to those used for the crosses, were available for one female in *S. diclinis* and *S. heuffelii*, 3 in *S. marizii*, 13 in *S. dioica*, and 17 in *S. latifolia* (Muyle et al. 2021). These samples were processed with the same cleaning, mapping, and genotyping pipeline as the cross data, except that we added the -par 1 option during genotyping to filter putative paralogs with paraclean. No comparable population-level transcriptomic datasets were available for *Artemia*, so no additional filtering step was applied for this case study.

### Estimation of synonymous divergence (dS)

To distinguish putative evolutionary strata, pairwise synonymous X–Y divergence (dS) was estimated for each sex-linked gene using module yn00 of the PAML program v4.10.7 (Yang 2007) with default parameters. For *Silene*, only exon sequences were retained, with UTRs and introns removed using custom R scripts. For *Artemia*, removing UTRs was not necessary, as they are not present in the reference genome annotation and are therefore absent from the generated sequences. Internal stop codons, common in degenerating Y- or W-linked genes, were replaced by “NNN” to prevent truncation artifacts in PAML. This ensured that dS estimates were calculated over full gene sequences rather than partial alignments.

### Allele topology analyses

Each pair of species—10 pairs for *Silene* and one pair for *Artemia*—was analyzed independently. Only biallelic SNPs were retained. In order to obtain the 14 possible topologies described in Fig. 1, it is necessary to use outgroups to orient them, i.e. to infer which allele is ancestral. For *Silene*, *Silene viscosa* served as the outgroup; we used RNA-seq data from three individuals sampled across different populations (Muyle et al. 2021). A consensus sequence was built after processing these reads with the same cleaning, mapping, and genotyping pipeline as for the focal species, and within-species SNPs were replaced by “N.” For *Artemia*, *Artemia franciscana* served as the outgroup; RNA-seq data from one female and one male were processed with the same pipeline to build a consensus sequence in which all sex-linked SNPs were replaced by “N.” Those data are available on NCBI under the project accession PRJNA449186 and sample accession SAMN19277832. If no ancestral allele could be inferred for a biallelic SNP in our focal species (e.g. because it was replaced with “N” in the outgroup), the corresponding topology was discarded.

Thus, once we know which allele is ancestral, we can encode each topology as four digits, assigning a value of 0 to the inferred ancestral allele and 1 to the derived allele. The first two digits represent the X and Y (or Z and W) alleles of the first species, and the final two digits represent those of the second species (see Fig. 1). We can then look at the relative frequency of each topology along the chromosome for each species pair to distinguish regions with a similar topological signature. If recombination suppression between the two sex chromosomes (X–Y or Z–W) occurred before speciation, the sex-linked haplotypes should cluster by chromosome across species, with all X (or Z) together and all Y (or W) together. If recombination persists, or if suppression occurred independently after speciation, topologies grouping the two sex chromosomes within each species are expected to dominate. Singleton topologies are also expected at relatively high frequency in all those regions (depending on terminal branch lengths, see Fig. 1), except in the PAR where the sex chromosomes should show very limited divergence.

Note however that no W reads were mapped to a large region at the right end of the Z reference chromosome for both species in *Artemia*, either because it is too divergent or due to a deletion. There are therefore no corresponding topology data on this part of the chromosome. The regions to the left and right of this hemizygous segment were analyzed separately because this segment already represents a specific part of the chromosome with its own history.

### Change-point analysis of strata boundaries

Change-point inference with an unknown number of change-points is often achieved by maximizing a penalized likelihood (Killick 2011; Killick et al. 2012). It requires specifying a probabilistic model for the data (a cost function), a search method, and a constraint on the number of change-points (the complexity penalty, Truong et al. 2020). We detail them in turn.

#### Cost function

We used the deviance of the data (−2 log-likelihood) as the cost function for both the analysis of dS and topologies change-point. In our case, topology data are categorical (14 possible states), and dS values are positive, strongly zero-inflated and right-skewed. They are observed for each sex-linked gene. We note $n_{G}$ the total number of sex-linked genes.

For change-point inference on dS values along the X/Z, we modeled observations with a zero-inflated gamma (ZIG) distribution, consisting of a point mass at zero with probability $\theta_{1}$ and a gamma distribution with shape $\theta_{2}$ and rate $\theta_{3}$ with probability (1–$\theta_{1}$). A model with *k* change-points partitions the chromosome into (k+1) segments, each with its own set of ZIG parameters. The location of the limits of each of those segments on the chromosome is stored in vector **v** of size *k*. For a segment *s* containing $n_{s}$ observations ($y_{i}$, $w_{i}$), where $y_{i}$ is the dS of gene *i* and $w_{i}$ a weight (the length in bp of the gene *i*, divided by the average gene length across all sex-linked genes), the log-likelihood of the data is:


(1) $$\begin{array}{rl} & \log\;L_{s}(y|\theta ,v)=\sum_{i=1}^{n_{s}}w_{i}\\ & \;\times \left\{ \begin{array}{cc} \log\;(\theta_{1}), & \text{if }y_{i}=0\\ \log\;(1-\theta_{1})+(\theta_{2}-1)\log\;(y_{i})-\frac{y_{i}}{\theta_{3}}-\theta_{2}\log\;(\theta_{3})-\log\;\Gamma (\theta_{2}) & \text{if }y_{i}>0 \end{array}\right. \end{array}$$

where Γ(.) is the Euler gamma function. Weights were used to account for the fact that the dS is better estimated for genes having a longer sequence.

For topology data, each observation falls into one of 14 possible states and is modeled with a multinomial distribution with probability vector $\theta =(\theta_{1},\ldots ,\theta_{13})$ [the proportion of the 14th topology $\theta_{14}$ is $1-\sum_{\;j=1}^{13}\theta_{j}$]. A model with *k* change-points divides the chromosome into (k+1) segments, each defined by its own probability vector. The location of the limits of each of those segments on the chromosome is stored in vector **v** of size *k*. For a segment *s* containing $n_{s}$ observed counts $c=(c_{1},\ldots ,c_{14})$, the log-likelihood is:


(2) $$\log\;L_{s}(c|\theta ,v)=\sum_{i=1}^{n_{s}}\sum_{\;j=1}^{14}c_{ij}\log\;(\theta_{ij}+e_{j})$$

where $e_{j}$ is a small constant equal to 0.01 if the topology *j* is a turnover topology, and 0 otherwise. This accounts for potential errors in the ancestral allele assignment. Indeed, such error could convert a singleton (e.g. 1,000) into a turnover (0111) topology. This error is specifically of concern. Indeed, turnover topologies are rare, with estimated *θ* very close to zero, and adding one such (very) unlikely observation by error can heavily impact the likelihood. The small error term $e_{j}$ prevents that error to have too much weight. Other topology counts would also be affected by errors in the ancestral allele assignment, but with much more limited consequences. Converting a singleton into a turnover topology will have very limited impact on the likelihood since the estimated proportions of singletons are large in all scenarios. For other topologies, swapping the ancestral allele does not change the per species (0011 and 1100) or per chromosomes (1010 and 0101) grouping patterns. Other errors in the dataset are possible but should be very rare. Indeed, to identify X/Y or Z/W alleles, we use crosses and parent—offspring data within the SEX-DETector pipeline. By sequencing several individuals and by checking the segregation of alleles, it becomes very unlikely to introduce errors in the allele identification.

#### Complexity penalty

To estimate the number of change-point and their location we minimized the function


(3) $$k(\left|\theta \right|+1)\log\;n_{G}-\sum_{s}2\log\;L_{s}$$

The second term is the sum over all segments *s* of the cost function defined in Equations (1) and (2) above. Note that the maximum likelihood estimates of $\theta_{i}$ in Equation (2) can be readily found as


(4) $$\theta_{i}=\frac{c_{i}}{\sum_{i}c_{i}}$$

The first term is the penalty, which is proportional to the number of fitted parameters (there are *k* segment limits in **v**, and |θ| parameters fitted within each segment) times the log of the total number of data point ($n_{G}$, the total number of sex-linked genes). This penalty corresponds to Schwartz information criterion (Schwarz 1978) and is commonly used in change-point analysis to severely prevent over-fitting (Killick et al. 2012; Truong et al. 2020).

#### Search method

Different methods have been used to perform change-point analyses (Truong et al. 2020). A commonly used method is the binary partition: a single change-point is exhaustively searched by minimizing the penalized likelihood for all possible locations. If this change-point is retained (as indicated by lower penalized likelihood than for the model with no change-point), then the method is iteratively repeated for the two sub-segments at the right and left of this first change-point location, and so on. This method is relatively fast but may not converge towards the optimal partition. Here, we used a more robust version: a *L*-partition. This was implemented using Mathematica 14.2 (Wolfram Research, Inc 2024). We first exhaustively search 1 to *L* possible change-points (considering all combinations of positions of change-points, with the constraint that each stratum must contain at least ten genes to avoid over-fitting). In models with more than three change-points, we use a quasi-exhaustive strategy: we first perform a coarse sub-sampling of candidate positions to identify promising regions of the chromosome and then refine the search locally around the highest likelihood peaks. If the lowest penalized likelihood was achieved for a number *l* of change-points lower than *L*, the estimated number of change-point was *l*. If the l=L change-point case had the lowest penalized likelihood, we then iterated the *L*-partition within each of the L+1 sub-segments identified. With L=1, the method is the same than the binary partition, but with L>1 it allows considering many more cases, limiting the risk of missing the right partition. In practice, the choice of *L* depends on the number of positions to consider (here the total number of genes) and the computing time available.

We used L=5 for topology data and L=2 for dS, as a good compromise to remain within 1-day computation time for one iteration on the whole sex-linked region with our data. Support limits for the final change-point locations were obtained from the set of contiguous locations within log $n_{G}$ units from the maximum, to stay consistent with Equation (3) (Casella and Berger 2002).

#### Detection and removal of outlier genes

After identifying regions with different topology proportions, we investigated whether some genes were outliers. Indeed, such outliers could occur for a series of reasons, and they could have a strong influence on the change-point analysis. For instance, outliers could occur because of a local error in the reference genome, or because of a very local rearrangement. To identify potential outliers, we searched for genes with topology counts that differed significantly from the estimated topology frequencies of their chromosomal region. To identify such outliers, we used a parametric bootstrap approach, in which observed likelihoods are compared to an empirical distribution generated by simulation under the fitted model.

Specifically, for each gene with m observed counts, belonging to a region with estimated multinomial frequencies θ, we resampled 20,000 draws from this multinomial with the same number of m counts. We then measured the proportion q of resampled counts with a lower log-likelihood than the focal gene. We did this for all sex-linked genes to obtain an overall distribution of log q for a given species pair. Cases whose observed log-q value fell at the extreme left tail of this distribution were identified as outliers. Cut-off values were set individually for the different species pairs, visually decided based on the discontinuity of the distribution on its left tail (typically at cut-off value between −6 and −5, see online supplementary material, Fig. S2). The whole change-point analysis was then rerun on the dataset without outliers to assess the robustness of inferred regions boundaries.

## Results

We first present the *Silene* and then the *Artemia* case.

### Silene

Using RNA-seq data on crosses analysed with SEX-DETector, we were able to describe for the first time the sex-linked regions of *S. diclinis*, *S. marizii* and *S. heuffelii* (see online supplementary material, Fig. S3).

#### Topology results

The change-point analysis based on topologies (Fig. 2) identified a large number of boundaries, ranging from ten to sixteen depending on the species pair. Many of these boundaries were shared across pairs. Based on the shared boundaries consistently present in all species pairs, broader regions were defined along the chromosome and labeled P, A1, A2, A3, L, and PAR in Fig. 2 and detailed below. Some gene outliers were detected (around 6% per species pair). Including or excluding them had a limited effect on the results. In most cases, one or two limits were removed per species pair when outliers were removed (online supplementary material, Fig. S4), but the positions of other limits were not affected. A summary of the limits of these regions is given in online supplementary material, Table S4. The raw topologies per gene are shown in online supplementary material, Fig. S5. To assist colorblind readers, a table with the proportion values for each topology in Fig. 2 is provided in online supplementary material, Table S2.

**Figure 2:**
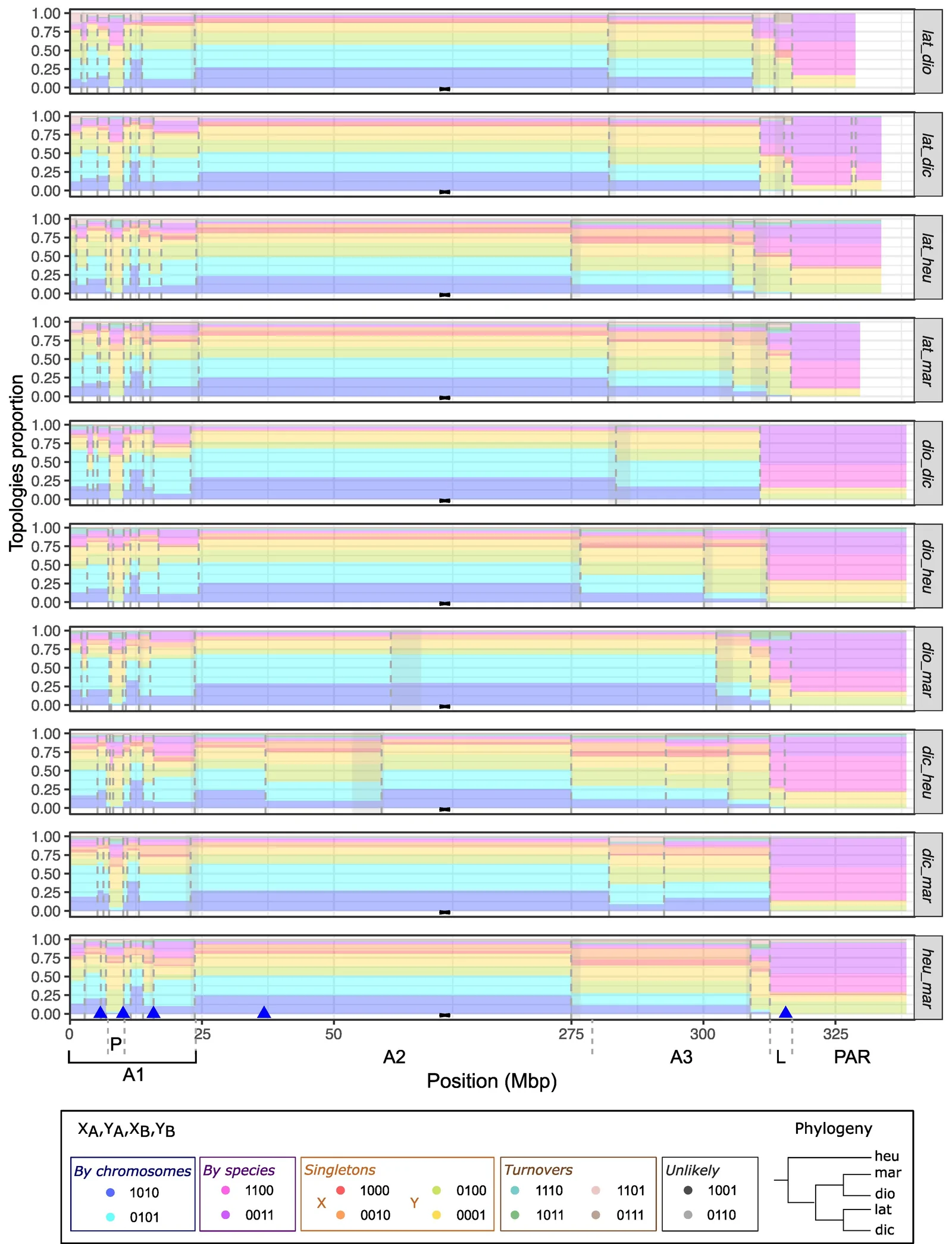
Regions inferred by the change-point analysis on topology proportions along the X chromosome for each pair of *Silene* species. The name of each pair is indicated on the right, abbreviated using the first three letters of each species’ name. Topologies are coded as in Fig. 1. Each dashed line represents a limit identified by the change-point analysis, with the respective support limits indicated by shaded grey rectangles around the limit dashed line. The figure indicates the results of the change-point analysis without outliers (see text). The results with outliers are illustrated in online supplementary material, Fig. S4. Blue triangles represent limits reported in the literature (see text). In each region, averaged topology proportions are shown as color stacks. The colors corresponding to each topology are indicated below the graph. Regions A1 to A3 have a high proportion of per chromosome topologies (blue and light blue).

#### Region-by-region patterns

Ancestral topologies grouping by chromosome were most prevalent in regions A1, A2, and A3 (where “A” stands for ancestral; blue in Fig. 2), alongside a substantial proportion of singleton topologies (red, orange, yellow, and green in Fig. 2). This pattern indicates that recombination suppression in these regions occurred before speciation in the ancestral species of the whole *Melandrium* section. Consistent with this observation, many of the limits of these regions are shared among the analyzed species. Y-linked singletons are more frequent than X-linked singletons across these regions, consistent with a faster accumulation of mutations on Y chromosomes. Region A1 was further subdivided into several small blocks compared to the rest of the chromosome. The region labeled PAR displayed many topologies grouping X–Y alleles by species (pink and purple in Fig. 2) and a smaller proportion of singletons. This region was interpreted as a pseudo-autosomal region (PAR). The singletons observed here, despite recombination, were likely false positives—genes identified as sex-linked due to the absence of crossing-over in the studied families. To try and circumvent this bias, we filtered sex-linked alleles using individuals from wild populations (please refer to Materials and Methods). *S. latifolia* and *S. dioica* had the largest wild population sampling. The fact that the proportion of singletons is lower in *S. latifolia* and *S. dioica* compared to other species confirms our interpretation that singletons in the PAR are mostly due to false positives. For species pairs involving *S. latifolia*, a region next to the PAR was detected as non-recombining. This region, labeled L for “latifolia specific”, exhibited a distinct pattern, with fewer per species topologies, more singletons, and some turnover topologies. Its consistent presence in all pairs involving *S. latifolia* suggests that it corresponds to an additional stratum that evolved specifically in *S. latifolia* (after the split with *S. diclinis*). The exact topological signature of this stratum, particularly the presence of up to 15% turnover topologies, remains however unclear. For *S. dioica–S. marizii* and *S. diclinis*—*S. heuffelii* pairs, a region next to the PAR was also detected. These regions were not found in other pairs involving these species, suggesting that it may correspond either to a weak signal or an artifact. A conspicuous region, labeled P for “puzzling”, was observed between positions 5.7 to 10 Mbp. Contrary to the surrounding regions that exhibit per chromosome ancestral topologies, this short interval consistently lacked such ancestral topologies and presented per species topologies. However, this region was consistently observed for all species pairs, with the same topological signature and the same boundaries, suggesting that it was already present in the ancestor. These two observations are difficult to reconcile. A possibility is that this region evolved shortly before speciation and was exposed to recurrent gene conversion afterwards.

#### Hypothesized evolutionary scenario

The delimitation and topological signature of the different regions can be used to propose evolutionary scenarios compatible with the observations, although this requires some interpretation and comes with uncertainties. As an example, we can propose an overall scenario that would match with the *Silene* case, insisting that it only represent the simplest possibility. All strata appear to be shared across the five species, except for an additional short stratum specific to *S. latifolia*. This implies that most evolutionary changes occurred in the ancestor of these species. A possible sequence of events is therefore as follows: Prior to species divergence, the ancestral sex chromosome evolved at least two initial non-recombining strata A1.1 and A1.2 with different ages. Secondary chromosomal rearrangements may have then subdivided this A1 region in small blocks. Additional strata (A2 and A3) subsequently accumulated. The small region P within the A1 region was likely present in the ancestor before speciation, but recurrent gene conversion in all species may have erased topologies by chromosome. Region P might either have evolved at the same time as region A1 (as represented in Fig. 3), or have resulted from a translocation from the X onto the Y in the ancestor, as proposed by Moraga et al. (2025). Finally, a more recent stratum evolved only in *S. latifolia*. A schematic summary of this tentative history is provided in Fig. 3.

**Figure 3:**
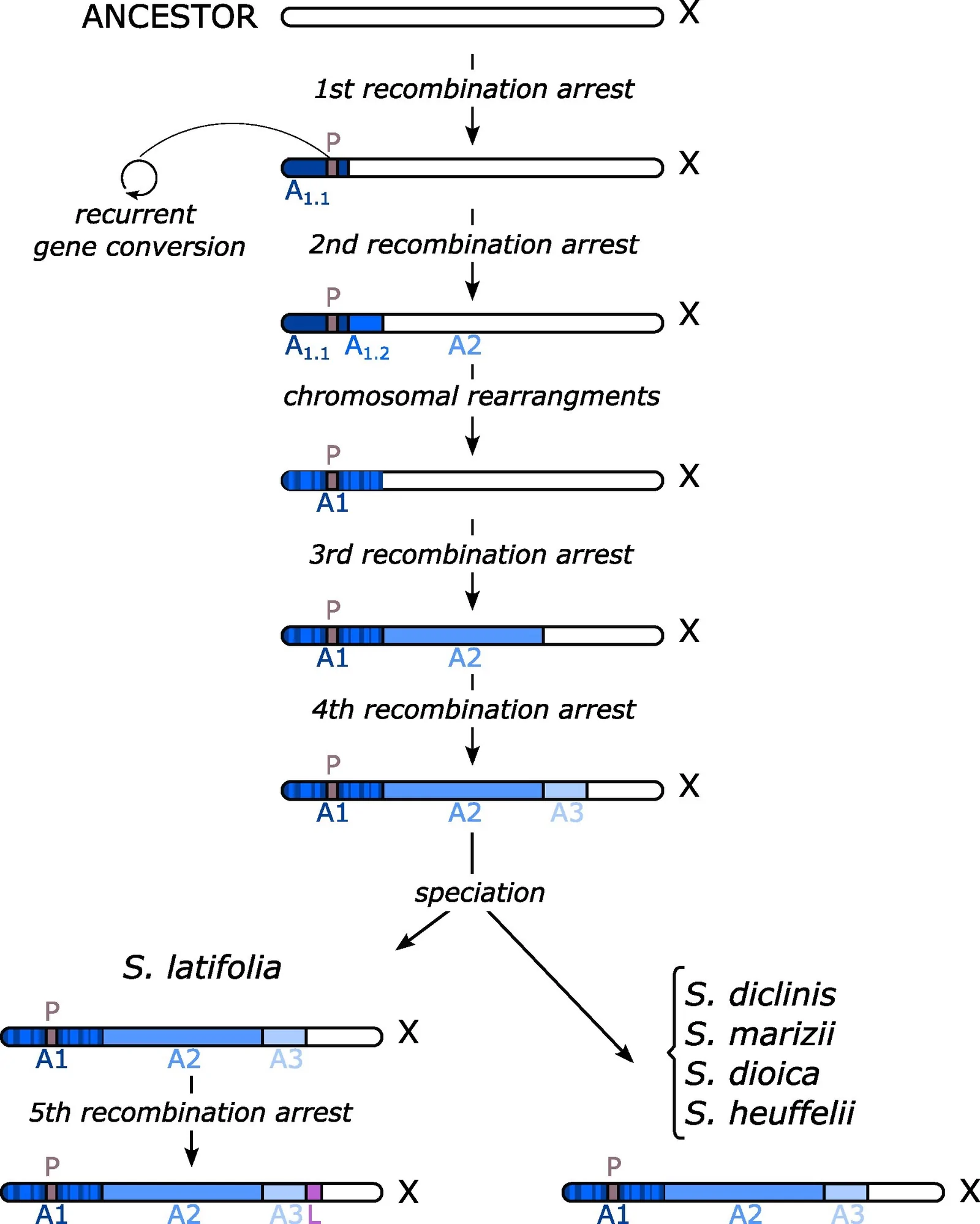
Schematic representation of one possible reconstruction of the events leading to the structure of sex chromosomes in the *Melandrium section*. This scenario is inferred using the change-point analysis on topologies (see text). For simplicity, the neo-sex chromosomes of S. diclinis are not represented. The ancestral strata (A1 to A3) are indicated in blue. The derived strata in S. latifolia is indicated in purple (L). The region shared by all species but showing per species topologies is indicated in gray (P). As explained in the text, this situation might be caused by recurrent gene conversion.

#### Comparison with dS data

Contrary to the topology analysis, only three to five change-points were inferred from the dS data, with three shared by all five species: the boundary delimiting the P region and the left limit of the PAR region (Fig. 4), although its exact position varied slightly. In *S. heuffelii*, it was shifted leftward, while in *S. latifolia*, it was shifted rightward, delimiting the additional small region labeled L. Overall, dS values decreased from left to right along the X chromosome, indicating that older strata are located toward the left end and more recent strata toward the right end, except for region P. dS values also fell near zero in the PAR region, consistent with the topology data. To facilitate direct comparison, a combined figure showing both dS and topology proportions is provided in the online supplementary material, S6.

**Figure 4:**
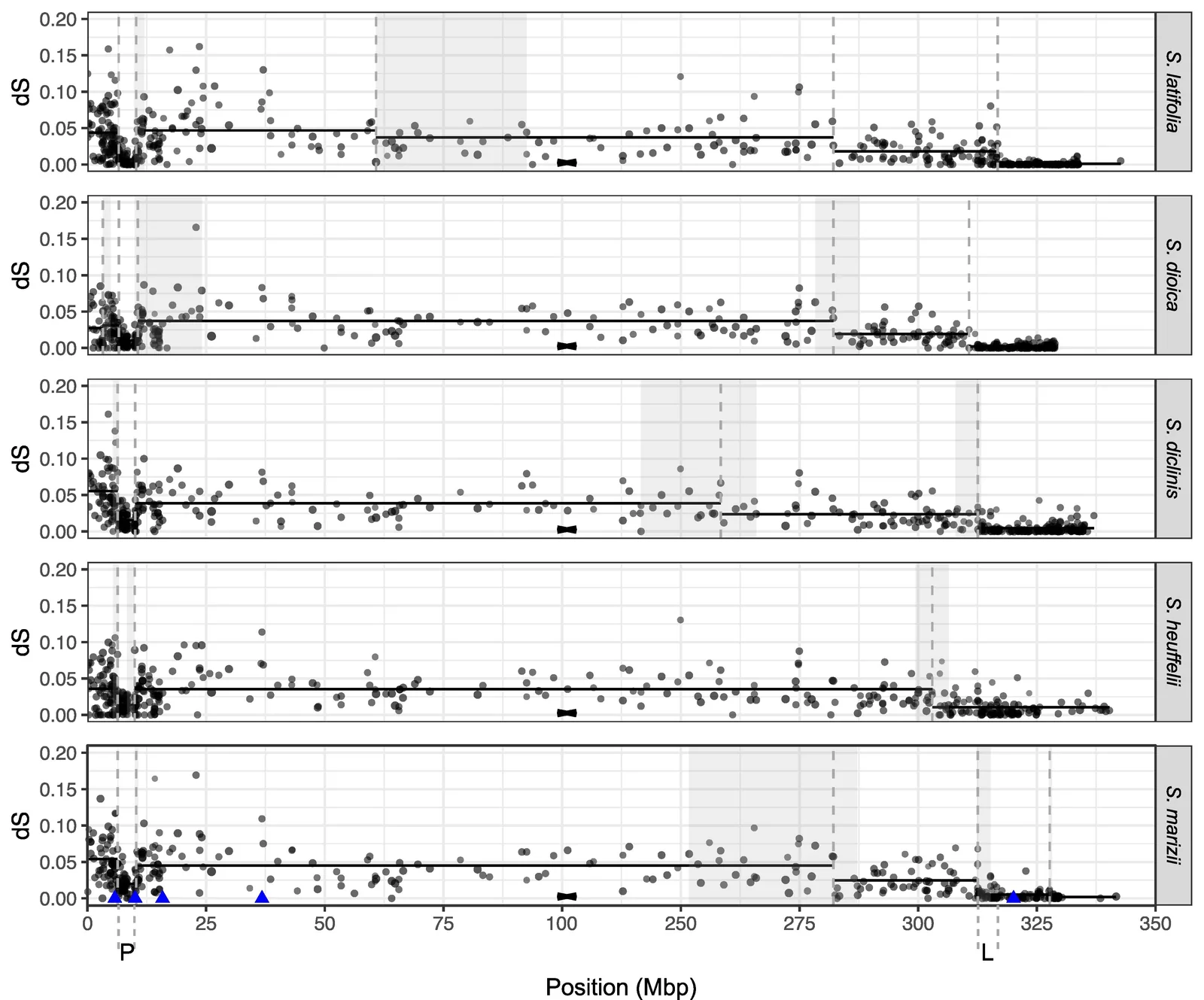
dS for each *Silene* species. Each dashed vertical line represents a limit identified by the change-point analysis on the dS, with the respective support limits indicated by grey rectangles around the vertical dashed limit. Blue triangles represent limits reported in the literature. Horizontal lines represent the average dS in each region. The name of each species is indicated on the right.

#### Comparison with the literature

The boundaries identified by Moraga et al. (2025), who produced the reference genome for *S. latifolia*, are represented as blue triangles in Figs. 2 and 4. Most previously described boundaries by Moraga et al. (2025) for *S. latifolia* were recovered in our analysis, in particular those flanking the P region (at 5.7 and 10 Mbp), the boundary at 15.8 Mbp (located inside A1 in our analysis), and the PAR limit at 315 Mbp. We did not find the 36.7 Mbp limit separating the “S1” and “S2” strata (as labeled in Moraga et al. 2025). We did find that the ancestral region was split in different parts. The closest corresponding limit would be at 24 Mbp, separating A1 and A2 in our notation. We also identified several other boundaries that were not described before, including the small blocks within the A1 region, and the left limit of region L, which delimits the additional stratum of *S. latifolia*. This additional stratum was mentioned by Filatov (2022), although precise boundaries were not published. A figure summarizing the comparison between the strata delimited with our method and those from the literature is available in the online supplementary material, S7.

### Artemia

Using RNA-seq data on crosses analysed with SEX-DETector, we were able to describe for the first time the sex-linked regions of *A. urmiana* (see online supplementary material, Fig. S8).

#### The hemizygous region

Due to the presence of a hemizygous region (no W reads mapped on the Z reference chromosome, see 1.6 of Material and Methods), the bordering genes were examined individually to avoid missing regional boundaries. Indeed, the limit of the hemizygous region may not exactly coincide with the limit of the non-recombining region that was removed. Small fragments may therefore flank the hemizygous region, that we could miss when focusing on large regions. To check for this possibility, we specifically looked at genes directly flanking the hemizygous region, to determine if they presented particular topological signatures.

At the left boundary of the hemizygous (region at 88 Mbp), such a gene—referred to here as G—was found. One part of this gene exhibited mainly per species and singleton topologies, while the other part displayed an ancestral signal, dominated by ancestral per chromosome topologies (blue in Fig. 5) alongside singleton topologies (red, orange, yellow, and green in Fig. 5). BLAST analyses revealed that this annotated gene in the reference genome was probably wrongly annotated and corresponds in fact to two genes, both encoding ribosomal subunit proteins (see online supplementary material, Fig. S9), the two genes match the two parts exhibiting the different topological signatures. This observation strongly suggests that one of those genes was present on an ancestral stratum that was almost entirely deleted from the W. Gene G location therefore represents a boundary between two regions with distinct evolutionary histories.

**Figure 5:**
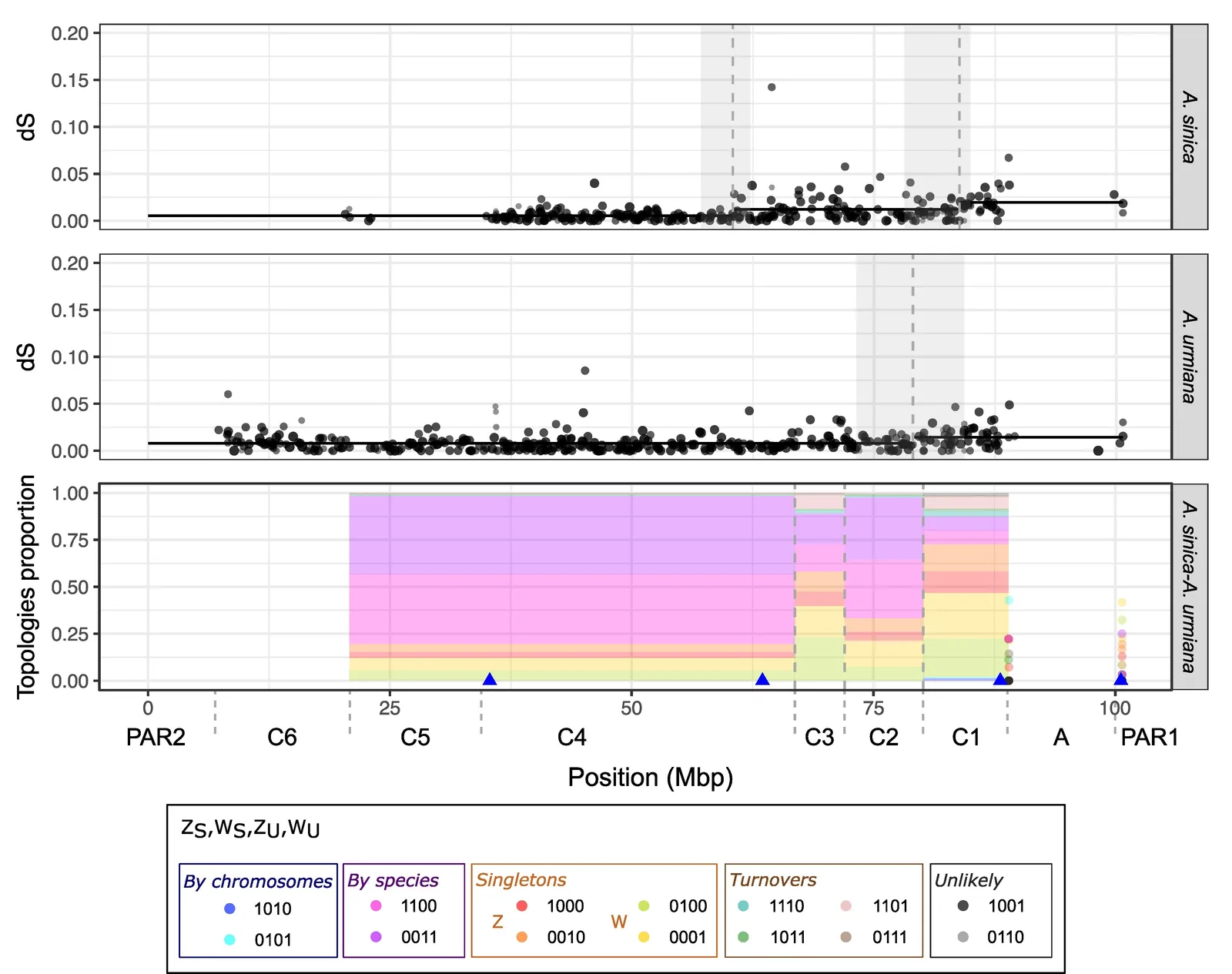
dS and topology change-point analysis for Artemia species. The analysis on the dS is shown on the two top panels and the topology analysis on the bottom panel. Codes are the same as in Figs. 2 and 4. Note however that the code for topologies is for Z and W instead of X and Y. The figure indicates the results of the change-point analysis without outliers (see text). The results with outliers are illustrated in online supplementary material, Fig. S11. The name of each species is indicated on the right. We observe that C1 to C5 regions have a high proportion of per species topologies. The C6 region corresponds to a region of the chromosome where A. urmiana has sex-linked genes but not A. sinica so that not topology analysis can be performed. The A region corresponds to the hemizygous region described in the text, with a single gene showing a per chromosome topology on its left limit (see the light blue dot).

At the right boundary of the hemizygous region (region at 100 Mbp), only two sex-linked genes were found. The two sex-linked genes primarily exhibited per species and singleton topologies, which could indicate either a non-recombining region that evolved independently in the two species, or a small additional pseudo-autosomal region (PAR). In the latter case, the presence of singleton topologies might result from the absence of recombination events in the studied families or from very low recombination rates in this region.

#### Region-by-region patterns

The analysis was performed on all genes located to the left of gene G. This analysis identified six non-recombining regions, labeled C1 to C6 in Fig. 5. The delimitation of these regions is based on two kinds of information: the change-point analysis and the occurrence of sex-linked genes in the two species. The part of the chromosome with sex-linked genes is much larger in *A. urmiana* than in *A. sinica*. We labeled C6 the region where sex-linked genes are detected in *A. urmiana* but not in *A. sinica*. Since allele topology analyses only include genes that are sex-linked in both species, no topological signal is available for this C6 region. We also distinguished C4 and C5 because of a strong discontinuity at 35 Mbp in the detection of sex-linked genes along the chromosome in *A. sinica* (see online supplementary material, Fig. S10). A cluster of five genes near 20 Mbp was indeed detected as sex-linked in *A. sinica*, although they may be false positives, or reflect an assembly error in the reference genome, given that other genes between 20 Mbp and 35 Mbp were identified as autosomal by SEX-DETector. It could also correspond to a species-specific rearrangement, given that the population from which our *A. sinica* samples originate is different to the one used for the reference genome. Removing outliers (see online supplementary material, Fig. S11) removed a limit at 65 Mbp delimiting a small region (65 to 67 Mbp). A summary of the limits of these regions is given in online supplementary material, Table S3. The raw topologies per gene are shown in online supplementary material, Fig. S10. To assist colorblind readers, a table with the proportion values for each topology in Fig. 5 is provided in online supplementary material, Table S2.

Examining the topology proportions in the C1 to C6 non-recombining regions identified above reveals that none of them seem to be ancestral, as they have a very low fraction of per chromosome topologies (that is why we labeled these regions with a “C” for convergent): region C1 has a very small fraction of per chromosome topologies (2.1%), and many more per species topologies (14.7%). In other regions, per chromosome topologies are absent (except in the 65 to 67 Mbp region mentioned above, where they account for around 1.4% if the outliers are kept). Strata C2 and C4 displayed very similar topological patterns, as did strata C1 and C3. This similarity suggests that a chromosomal rearrangement, such as an inversion, occurred between regions C2 and C3 in either *A. urmiana* or *A. sinica*. Indeed, the *A. sinica* strain used in our study and the one used to create the reference genome come from two different places (Tanggu salterns and Xiechi Lake respectively, both in China), so it is possible that the observed chromosomal rearrangements happened only in the Xiechi Lake strain. In strata C2 and C4, the dominant topologies grouped chromosomes per species, with a substantial proportion of singleton topologies, consistent with independently evolved non-recombining strata in the two species. In contrast, strata C1 and C3 showed higher proportions of singleton, turnover, and unlikely topologies. This complex signature arises from highly heterogeneous genes within these regions. Most genes exhibited a simpler pattern with per species and singleton topologies, while a few genes displayed a mixture of turnover, per chromosome, per species, unlikely, and singleton topologies, encompassing all possible topologies. This suggests a more complex evolutionary history in these regions.

#### Hypothesized evolutionary scenario

Like in the *Silene* case, these observations allow us to propose a plausible minimal scenario (Fig. 6). First, recombination suppression occurred in the ancestor of *A. sinica* and *A. urmiana* (before the split with *A. franciscana*, following Elkrewi et al. 2022), forming an ancestral stratum. This stratum was then either mostly deleted before speciation or accumulated so much divergence that the Z and W were no longer homologous in this region. The similarity of regions C1-C3 and C2-C4 suggests that a secondary rearrangement, such as an inversion, occurred in one of the two species that had two strata of different age. In the other species, at least one non-recombining region independently evolved. However, the topological information alone does not indicate which species is which. Figure 6 illustrates the case where *A. sinica* is the species with the two strata (labeled S1 and S2 in the figure) and the inversion. In that minimal scenario, *A. urmiana* would have just one independently evolved stratum. This stratum would be larger than the size of S1 and S2 combined (the extra part corresponding to the C6 region described above).

**Figure 6:**
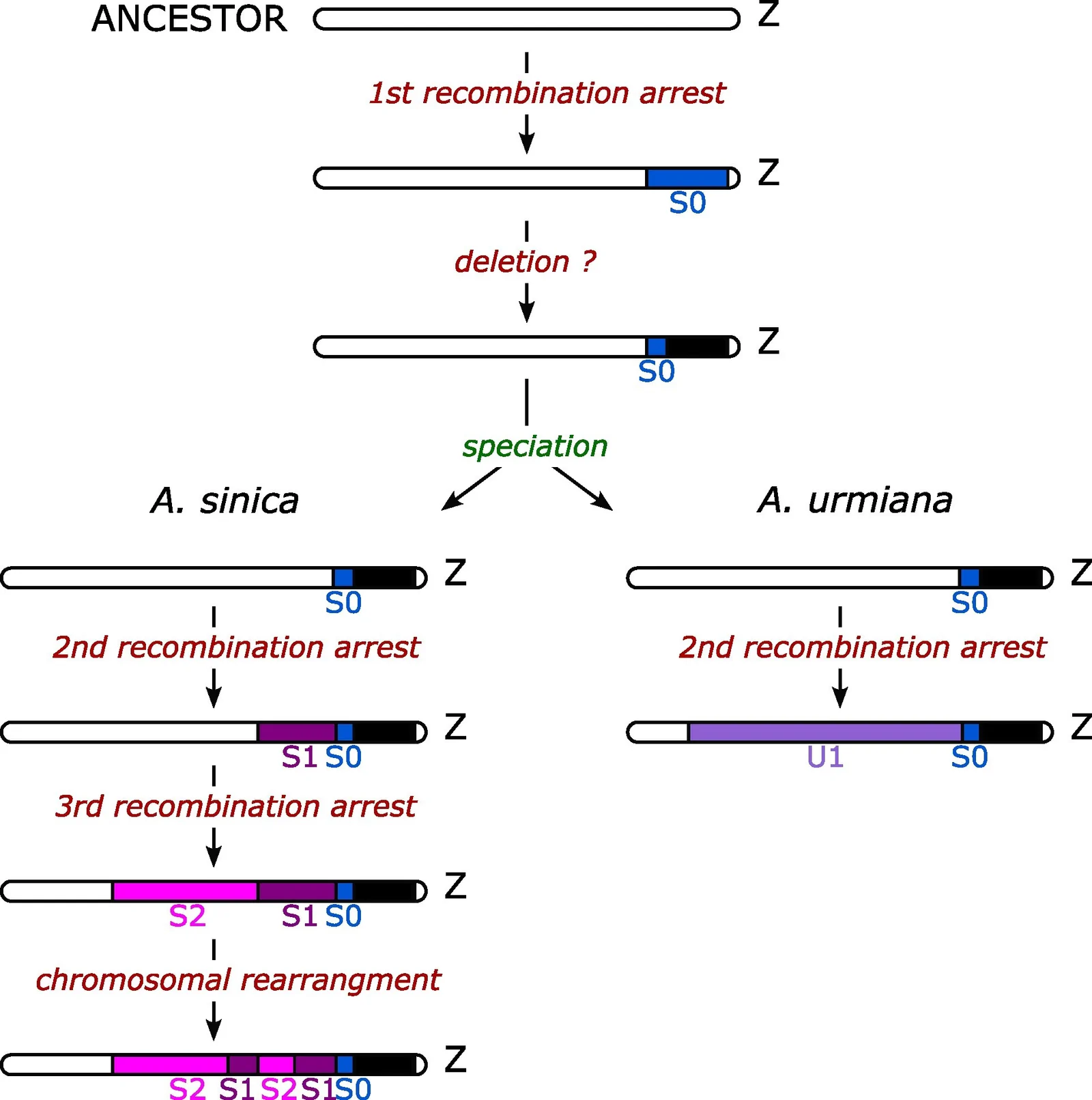
Schematic representation of one possible reconstruction of the events leading to the structure of sex chromosomes in *A. sinica* and *A. urmiana*. This scenario is inferred using the change-point analysis on topologies (see text). The ancestral stratum is shown in blue (S0) and the hemizygous region is shown in black and may correspond to a deletion. The derived strata in each species are indicated in pink for A. sinica (S1 and S2) and purple in A. urmiana (U1). The scenario illustrates a secondary rearrangement occuring in A. sinica between S1 and S2 leading to the four sub-regions in that species (C1 to C4 in Fig. 5).

#### Comparison with dS data

The dS analysis was less precise than the topology analysis, with fewer limits and wider support limits (which is analogous to a confidence interval). Only two boundaries were detected in *A. sinica* (60 and 83 Mbp) and a single boundary (79 Mbp) in *A. urmiana* (Fig. 5). The limits in the two species close to 79 to 83 Mbp might correspond to the C1-C2 limit at 80 Mbp identified by the topology analysis. The 60 Mbp limit found in *A. sinica* might correspond to the 67 Mbp C3-C4 boundary, although it is shifted leftward. This difference may result from the inclusion, in the dS analysis, of the outliers in the 65 to 67 Mbp region detected with topologies. The fact that the dS analysis detects more limits in *A. sinica* than in *A. urmiana* suggests that the species with the secondary inversion mentioned in the scenario above is more likely to be *A. sinica*. However, the detection of a dS limit in *A. urmiana* suggests that there may be more than just one independently evolved non-recombining stratum in this species. This would slightly complicate the scenario proposed above. It is possible indeed that the limit of this extra *A. urmiana* stratum (around 79 Mbp as indicated by the dS) is too close to the C1-C2 limit and therefore delimit a region that is too small (less than 10 genes) to be detected in the change-point analysis.

#### Comparison with the literature

The boundaries identified by Elkrewi et al. (2022), who produced the reference genome for *A. sinica*, are represented as blue triangles in Fig. 4. Most previously described boundaries were recovered in our analysis. Those at 88 and 100 Mbp, which delimit the ancestral strata labeled “S0” in Elkrewi et al. (2022), roughly correspond to our heterozygous region plus half of gene G. We have therefore also labeled this region S0 in our hypothetical scenario (Fig. 6). The boundary identified by Elkrewi et al. (2022) at 35 Mbp matches the C4-C5 limit in our case. There are nonetheless few differences. The boundary they identified at 63 Mbp may correspond to our C3-C4 limit at 67 Mbp (but see above the issue of outliers in the 65 to 67 Mbp region). We identified additional limits: the C1-C2 and the C2-C3 limits.

## Discussion

### A new method with improved precision and sensitivity

Our topology-based approach has proven effective in detecting both recombination arrests (primary events, e.g. inversions on the X or the Y) and rearrangements on the X (secondary events) in sex chromosomes. For both *Silene* and *Artemia*, we successfully recovered almost all previously described boundaries, validating the reliability of our method. The separate analysis of all species pairs in the *Silene* case also demonstrated the robustness of the method, as most limits in ancestral regions were shared among all species pairs. Furthermore, we identified additional boundaries and likely chromosomal rearrangements—such as the secondary rearrangement in *Artemia* and multiple rearrangements in the ancestral strata of *Silene*—that were not clearly resolved in earlier studies. These findings suggest that the evolutionary histories of these groups are more intricate than previously assumed.

When compared to dS-based analyses, our topology-based method provides several advantages. First, it yields more precise boundaries, with narrower support limits, allowing for a clearer separation of strata. Second, it is more sensitive, enabling the detection of a greater number of limits with clear different topological signatures. Third, the topological signal is more informative, as it captures fine-scale patterns that single summary measures of divergence (such as dS, Fst, or dXY) may lack the resolution to detect. This allows us to directly propose evolutionary scenarios. We can identify which strata are ancestral and which are convergent, and identify regions with similar topological signature (e.g. the C1-C3 and C2-C4 comparison in the *Artemia* case). However, the method still presents some limitations that we detail below.

### Addressing the challenges of a sensitive method

While the increased sensitivity of our method is a significant strength, it also introduces challenges that require careful consideration. First, there is a risk of over-fitting and over-segmentation. Defining strata boundaries inherently involves a trade-off between over-fitting and underfitting. Overly sensitive methods may split regions at the gene scale, potentially creating artificial subdivisions, while overly coarse approaches may miss important evolutionary events. This balance is particularly critical in change-point analysis, where the risk of over-segmentation is well-documented (Killick et al. 2012; Truong et al. 2020; Zou et al. 2020). In our study, we addressed this challenge by imposing a minimum number of ten genes per stratum and implementing an outlier detection procedure. Genes with extreme or ambiguous signals were removed, and the analysis was rerun to ensure robustness. Comparing results with and without outliers showed minimal changes, indicating that our method remains reliable despite some noise in the topology data. This robustness is partly due to the high quality of our sex-linked allele detection, which relies on segregation patterns in families with at least four offspring of each sex. Note that alternative pipelines can be used to recover these sex-linked alleles (see Introduction and Materials and Methods). Genes with ambiguous reads or potential errors were systematically excluded. However, the quality of the outgroup—less robust than the X/Y or Z/W sequences—can still introduce errors in ancestral allele assignment. For instance, a misassigned ancestral allele could convert a singleton topology into a turnover topology, or vice versa. To account for this uncertainty, we included an error parameter in the change-point analysis for these specific topologies. Despite these precautions, we may still overfit the data. This can be observed in the *Silene* case, where few limits where specific to one species pair comparison and made little sense with respect to the phylogeny of the *Melandrium* section. For example, in the comparison between the distantly related species *S. diclinis* and *S. heuffelii*, the A2-A3 region is divided into several subregions with similar topological signatures. However, these subregions are not present in other comparisons, even those involving these two species. Hence, these limits should be treated with caution. It is difficult to determine whether these boundaries are false positives in the species pair where they were detected (resulting from over-fitting or from the limitation of the L-partition method to find the maximum likelihood), or if we simply lacked the power to detect them in other pairs. This shows how having a well-established phylogeny can be particularly useful, as it allows us to check post hoc whether boundaries appear consistently across all relevant species pairs or whether not. Overall, our method seems to achieve a good compromise between sensitivity and robustness, as it recovers almost all previously described strata, including the very young *S. latifolia*-specific stratum, as well as new boundaries consistent across species pairs.

Second, with our data, we cannot always confidently determine whether a region interpreted as a chromosomal rearrangement truly represents a biological event or an assembly error. Both scenarios can produce similar topological signals, especially since all species are mapped to the X or Z chromosome of a single reference genome. One potential solution would be to use reference genomes for each species and perform synteny analyses. However, this approach is often impractical, particularly for sex chromosomes, which are notoriously difficult to assemble (Tomaszkiewicz et al. 2017; Carey et al. 2022).

Third, the detection of secondary events, while valuable, can complicate the interpretation of evolutionary histories, especially in ancient strata where multiple events may have occurred. In *Silene*, for example, while ancestral strata are relatively easy to identify due to their distinct topological signatures, they can be further complicated by rearrangements on the X. Our analysis showed that what we labeled as region A1 is subdivided, making it difficult to pinpoint exactly where the first recombination arrest occurred. This complexity arises from X rearrangements that probably occurred differently in the different species after speciation. However, the situation would be even more challenging if we had to also account for the potential presence of Y secondary rearrangements. The obtention of high-quality X and Y assemblies will enable synteny analyses to directly compare the structure of the chromosomes and identify some structural breakpoints. While synteny analyses can identify structural blocks, they provide no information about their evolutionary history, making it extremely challenging to reconstruct a clear historical scenario, especially when numerous structural changes have occurred. So, even if the X and Y chromosomes are assembled, it would still be valuable to analyze topology patterns as we propose. By computing the proportions of topologies after aligning SNPs to a single reference, one can filter out secondary rearrangements occurring on the non-reference chromosome (e.g. Y if X is the reference) and infer some possible scenarios for recombination suppression. Additionally, the topological signature can help identify regions that share the same evolutionary history, although physically separated by secondary rearrangements occurring on the reference chromosome (e.g. the X if X is the reference). Since such regions would exhibit very similar proportions of topologies, our method makes it easier to group them and help infer the evolutionary scenario. Thus, our approach helps to identify the primary steps of recombination suppression and to compare the delimited blocks with the structural breakpoints identified independently using X and Y assemblies.

### The challenge of PAR annotation

Our analyses of *Silene* and *Artemia* underscore the difficulty of reliably estimating the boundaries of the PAR. We identify sex-linked genes using crosses, with parents and offspring data. This strategy has the advantage of almost entirely removing wrong X–Y or Z–W misassignment and removes most issues related to sequencing and genotyping error. However, in a given cross, the closest crossover to the PAR boundary may still be at some distance to that boundary. Hence, we could detect genes that are sex-linked in the dataset but are actually located in a recombining portion of the chromosome. One way to get around this problem is to use additional population data, as we did for *Silene*. We observed that, indeed, using population data clarified the topological signature in this area (see online supplementary material, Fig. S12). In species pairs involving *S. latifolia* and *S. dioica*, the two species with abundant population data, we observed fewer singleton topologies in the region at the right from A3 after the correction. The population correction also removed much of the dS variation in this area (online supplementary material, Fig. S12). Yet, including this population data did not change much the length of the region harboring sex-linked genes. Indeed, no sex-linked genes were entirely removed with the population correction, as they all retained at least one sex-linked SNP not identified as a false positive by the population data. The PAR was therefore identified as the region with few singleton topologies and dS near zero, despite the fact that it corresponds to a region with sex-linked genes in the dataset.

In *Artemia*, where we did not have population data, we did not observe any region with sex-linked genes with few singletons that might be interpreted as a region between the true PAR limit and the position of the last crossover in the cross dataset. It is therefore likely that the last crossover in the cross dataset was close to the PAR boundary, implying a high recombination rate in the PAR. The other possibility could be that all crossovers in the crosses are very far from the PAR boundary and that the entire region left to C3 is within the PAR. However, this is unlikely, as the C3-C4 limit was independently found by Elkrewi et al. (2022) on a different *A. sinica* strain than the one used for this study.

From these observations, we conclude that the population correction can improve the signal (for the topologies and the dS), but does not entirely address the issue of positioning the PAR boundary, despite representing a large effort in terms of data acquisition. The limit of the region with sex-linked genes did not change much after this correction. Beyond the examples of *Silene* and *Artemia* presented here, these PAR boundary uncertainties reflect a broader challenge in sex chromosome research, as highlighted by the guppy debate (Wright et al. 2017; Bergero and Charlesworth 2019; Darolti et al. 2020; Kirkpatrick et al. 2022). It is difficult to distinguish regions where recombination has recently stopped from regions where recombination still occurs but at very low rates. In both cases, some X–Y divergence can accumulate, producing similar signals. Consider a region that appears non-recombining due to accumulated divergence. If this region is young (e.g. 100,000 years), we are sampling a relatively narrow time window. In this case, the observed divergence may simply reflect the absence of crossovers during this period, even if recombination could still occur in the future. Now, imagine observing the an older divergent region, such as one dated to 10 My. The probability that this region is actually recombining but no crossover occurred by chance over 10 My becomes extremely low, even if the recombination rate is very low. Thus, it is less likely to falsely interpret such a region as non-recombining. Therefore, one should always consider the sampled time window—that is, the evolutionary timescale being examined—and keep in mind that the absence of evidence for recent recombination is not definitive proof of recombination suppression, regardless of the method used. Furthermore, recombination suppression is not necessarily permanent; it may vary among lineages or be restored in the future through turnover or structural changes.

### From strata identification to evolutionary scenarios

As we have shown, our method may help building plausible scenarios for reconstructing the history of changes occurring in the non-recombining regions of the sex chromosomes. Figures 3 and 6 illustrate this. However, it is important to note that our method does not provide direct insight into the mechanisms that caused recombination suppression. While we can infer when and where recombination stopped, determining how it happened would require additional data or approaches, such as reliable Y assemblies. Additionally, our method cannot infer scenarios for regions where either the X or Y sequence is missing. As seen with the S0 region in *Artemia*, if a reliable Y sequence cannot be obtained for a given region—either because the sex chromosomes are too divergent for the Y to map correctly to the X reference (technical issue) or because there is a deletion on the Y (biological issue)—the method cannot be applied due to missing data. This limitation is shared with other divergence-based methods, such as dS, Fst or dXY. In such cases, male-to-female coverage depth analysis may be more appropriate. Furthermore, the method is primarily designed to identify regions of the chromosomes sharing a common evolutionary history, but not to test statistically among different scenario. This would represent an interesting perspective of this work, as, in principle, it should be possible to map a given scenario to a topological signature and build likelihood models to infer the most likely ones. Such an approach is beyond the scope of this paper, and probably represents a significant challenge, as those scenarios can be quite complex, or at least more complex than usually presented. Our case studies demonstrate this complexity, with the likely occurrence of few strata and secondary rearrangements, already creating several regions with a different signature. If this method was extended to statistically compare for evolution scenarios, another difficulty would be that scenarios might not be entirely clear. For instance, in the *Artemia* case, in the C1 and C3 regions, the topological signature is complex with all possible topologies present. It cannot be a turnover event or an introgression because both would only create one kind of turnover topologies. Instead, we observed all of them and also unlikely topologies. Such observations might be caused by a diversity of phenomena, including incomplete lineage sorting or gene trafficking. They probably need to be better modeled. In the *Silene* case, region P previously identified in *S. latifolia* was found to also exist in all five species of the *Silene Melandrium* sections and we were able to refine its boundaries. However, the topological signature of region P—lacking ancestral topologies despite being found in all species pairs with the same limits and thus probably ancestral—remains puzzling. Moraga et al. (2025) showed that this small region did not result from a genome assembly error, as it was present and syntenic on the homologous chromosome of the outgroup species *S. conica*. As mentioned above in the proposed scenario for *Silene*, it may be possible that this region experiences gene conversion at a high rate (compared to surrounding ancestral regions) in all species. Some genes on sex chromosomes seem indeed to be hotspots for gene conversion (Rosser et al. 2009; Trombetta et al. 2014; Wright et al. 2014; Peneder et al. 2017), but here, it would require that this hotspot extends to a specific 4.3 Mbp region.

## Conclusion

Our method is primarily designed to help identifying strata on sex chromosomes, beyond the techniques that have been used so far. We showed that it provided novel and robust findings, on existing case studies. It can apply to a wide range of biological cases, as shown by the different examples that we used here. It needs however to be combined with existing other methods. In particular, the *Artemia* case shows that extra information, on the dS, or the Fst helps to better understand the situation, stressing that combination of information and approaches remains critical to study sex chromosomes.

## Supplementary Material

msag249_Supplementary_Data

## Data Availability

Sequencing data for *Artemia* species are available in the NCBI under project PRJNA1530745. All custom scripts and code used for RNA-seq data analysis and figure generation are available on GitHub: https://github.com/suzie-tallon/topology_based_sex_chromosome_partition.
