# Supplementary material for: Using gene topologies to partition sex chromosomes into regions with distinct histories
Source: Mol Biol Evol. 2026 Oct 16;43(10):msag249. doi: 10.1093/molbev/msag249 (PMC13673985; doi:10.1093/molbev/msag249)
Supplement: Supplementary file 1 [file msag249_supplementary_data.zip › Supplementary_Data.pdf]

Supplementary Data

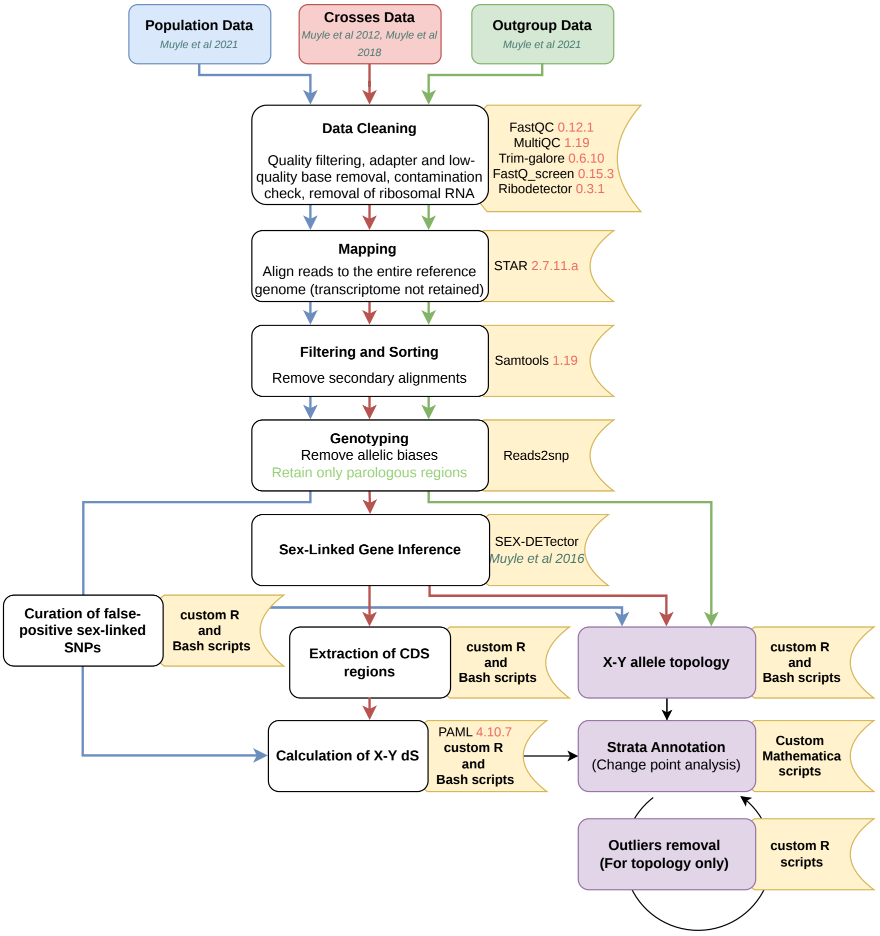

Figure S1 Pipeline of all the programs and scripts used for the various stages of data processing. *The steps that can be performed using aligned X and Y alleles from another pipeline are shown in purple.*

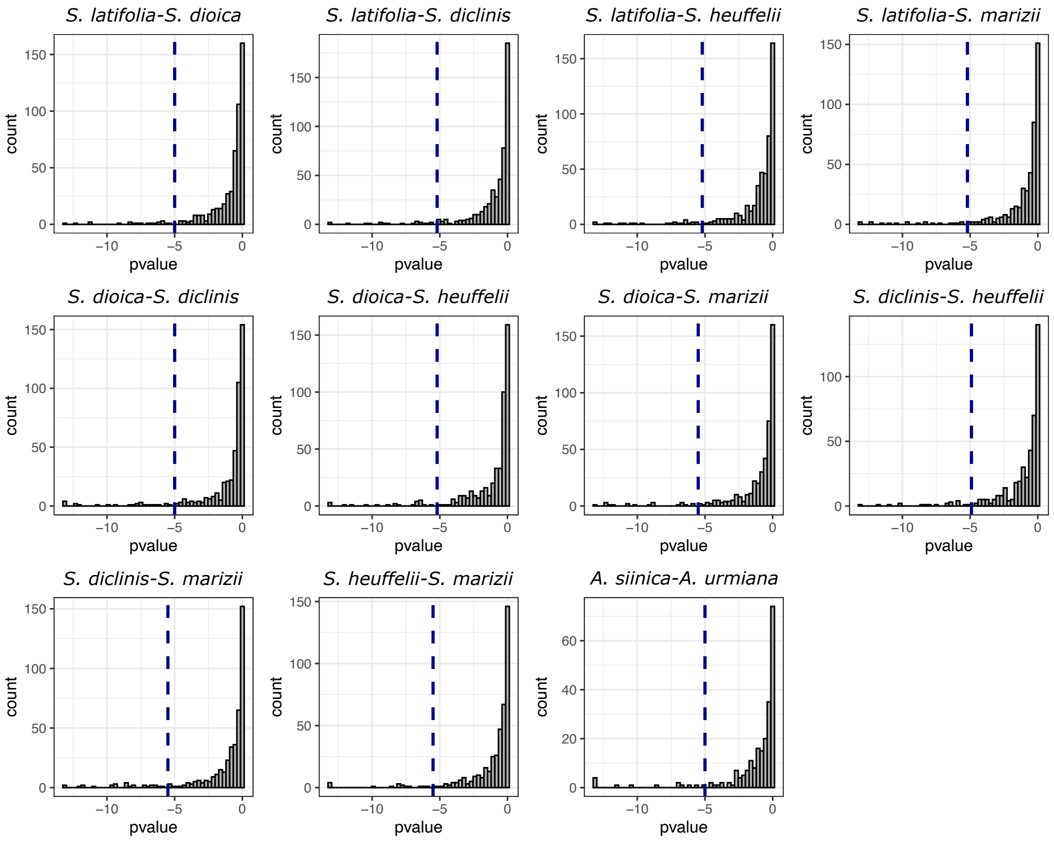

Figure S2 Outlier analysis. *The histograms show the distribution of log q (the proportion of re-sampled counts with a lower log-likelihood than the focal gene) for each pair of Silene species (see methods). The dashed lines indicate the cut-off values that were used.*

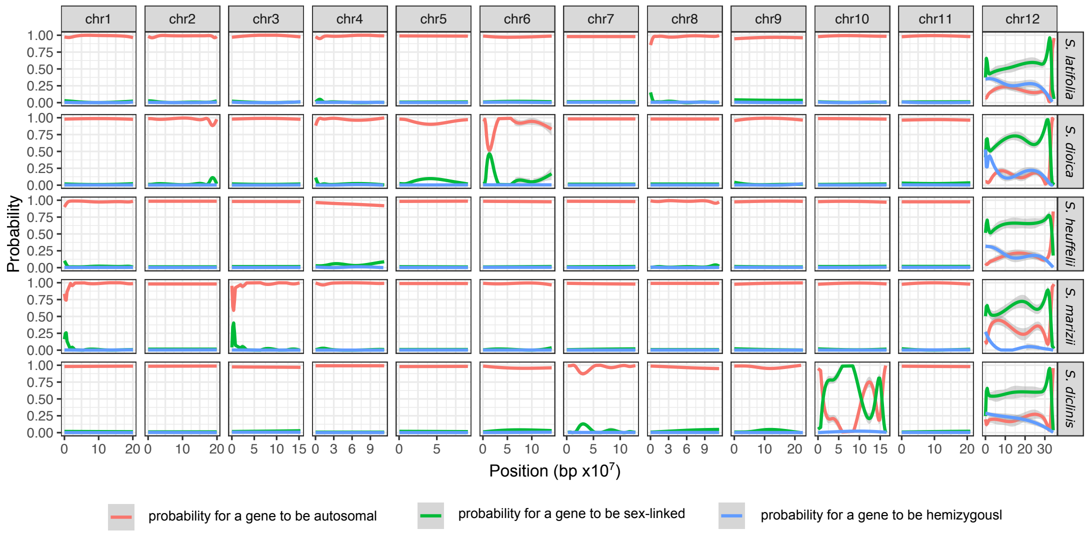

Figure S3 SEX-DETector analyis for *Silene*. *The figure shows the probabilities for each gene to be autosomal (red), sex-linked (green) and hemizygous (blue) for all chromosomes and all species. The result are smoothed using a sliding window averaging (using geom_smooth of* `ggplot2` *package). Chromosome* 12 *in S. latifolia is also the sex chromosome in the other four species. These results also confirm the presence of a neo-sex chromosome in S. diclinis (Howell et al. 2009) resulting from a translocation between the ancestral Y chromosome and an autosome, identified here as chromosome 10. Additionally, small sex-linked regions were detected outside of chromosome* 12 *in S. dioica and S. marizii, suggesting that rearrangements may also have occurred in these species. Genome assemblies of these species will be necessary to confirm whether these regions are truly sex-linked or false positives.*

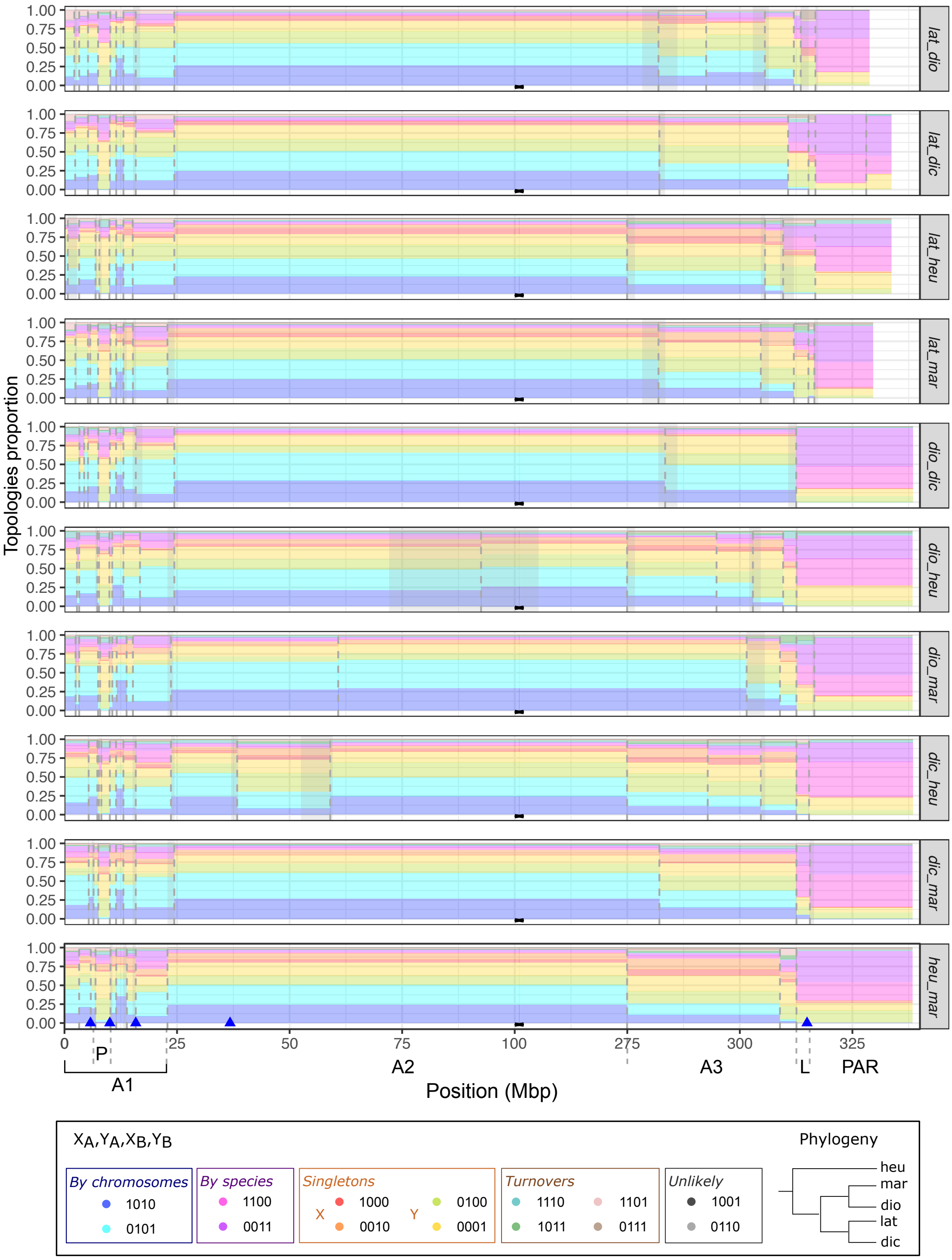

Figure S4 Regions inferred by the change-point analysis on topology proportions along the X chromosome for each pair of *Silene* species. *Same as Fig. 2 but showing results including the outliers.*

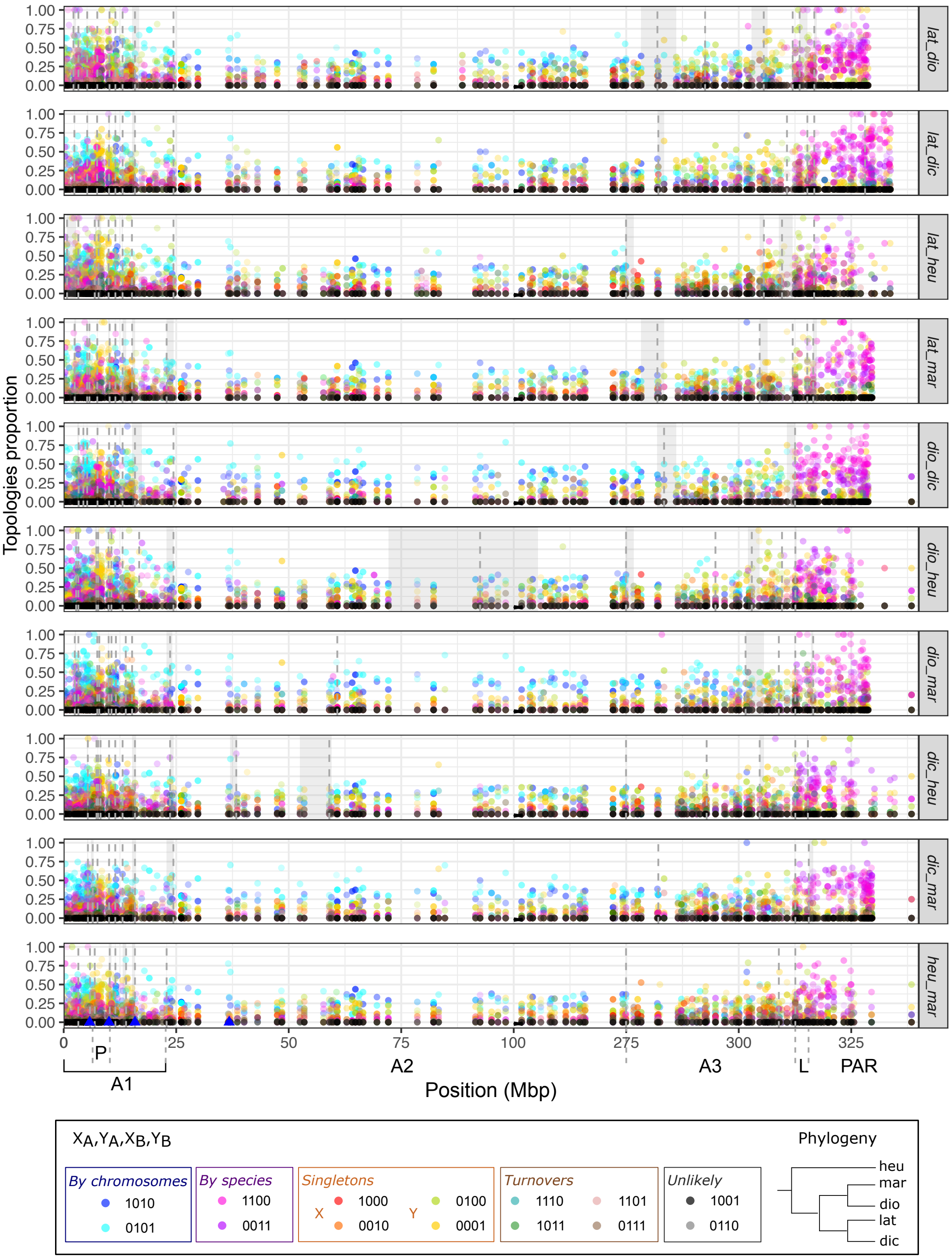

Figure S5 Raw topologies for *Silene* species pairs. *Same as Fig. S4 but with raw topologies per gene instead of per region averaging.*

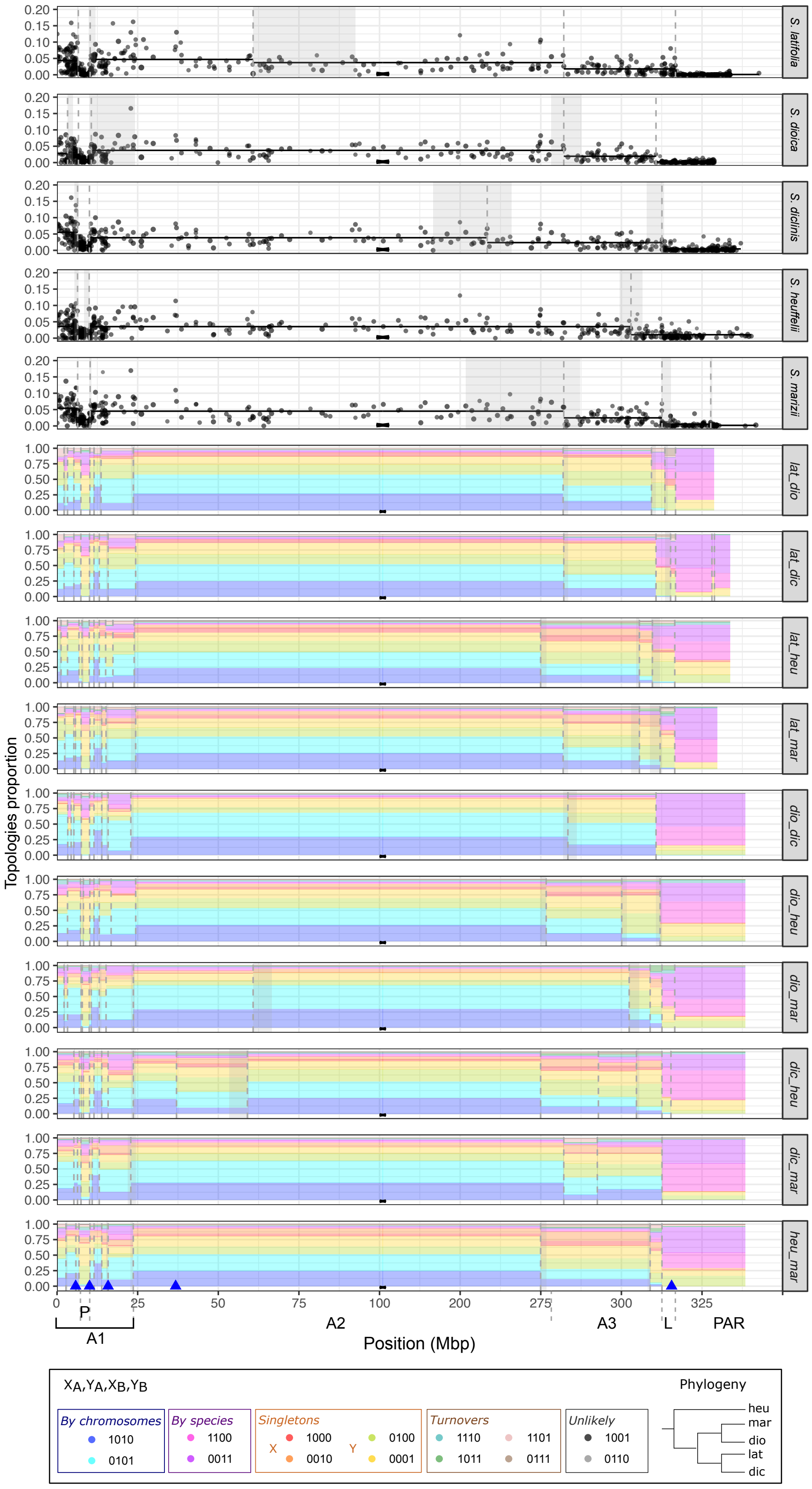

Figure S6 Combination of Figs. 2 and 4 for easier direct comparison.

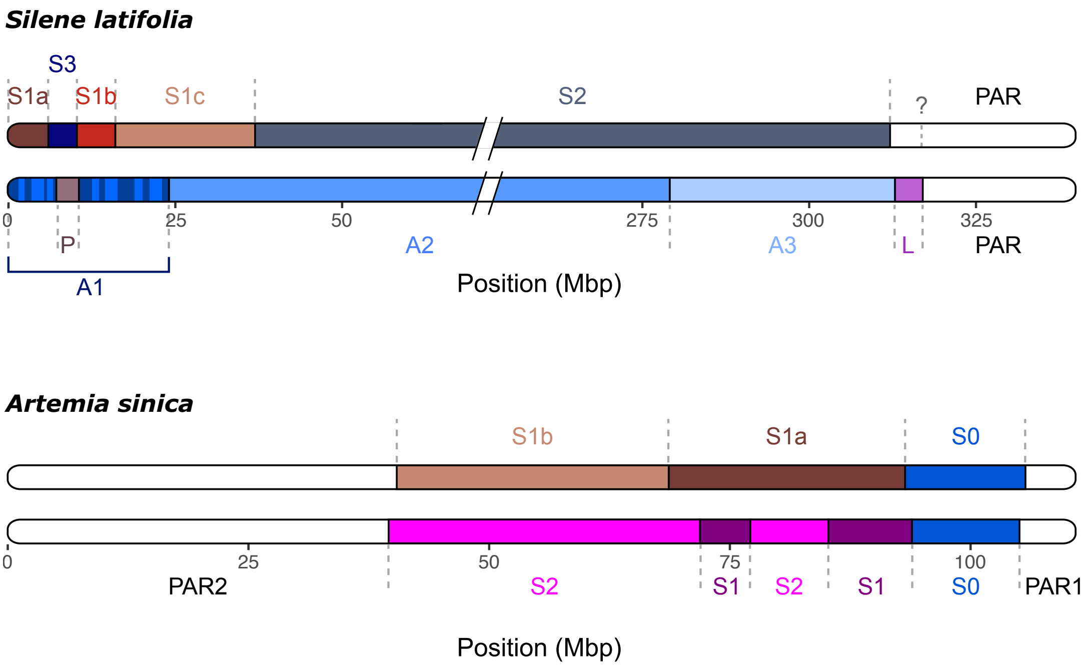

Figure S7 Comparison between our results and the literature for *Silene latifolia* and *Artemia sinica*. *For each species, two chromosomes are shown: the top one indicates strata from the literature with their original names, and the bottom one shows our results with our notation. This allows a direct comparison between the two sets of notations. For S. latifolia, colored strata are from Moraga et al. (2025); the limit found by Filatov (2022) is shown in gray with a question mark, as its exact location was not provided. For A. sinica, literature strata are from Elkrewi et al. (2022).*

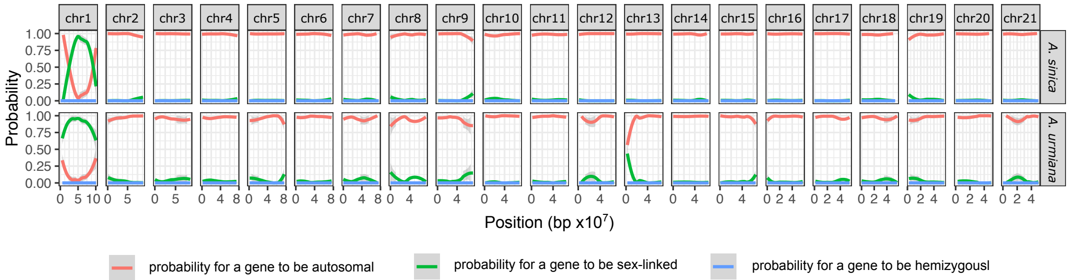

Figure S8 SEX-DETector analyis for *Artemia.The figure shows the probabilities for each gene to be autosomal (red), sex-linked (green) and hemizygous (blue) for all chromosomes and all species. The result are smoothed using a sliding window averaging (using geom_smooth of* `ggplot2` *package). Analysis of the RNA-seq data using SEX_DETector revealed that, as in A. sinica, chromosome* 1 *is the sex chromosome in A. urmiana. A small region at the beginning of the* 13*th chromosome may also be sex-linked in A. urmiana. Genome assembly of that species will be necessary to confirm whether that regions is truly sex-linked or a false positive.*

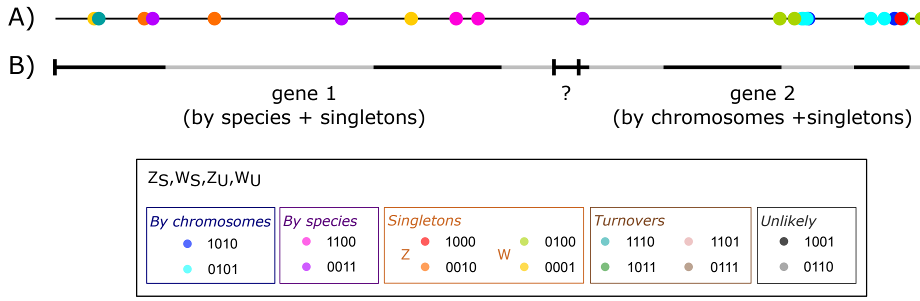

Figure S9 Specific analysis for G gene. *As explained in the text, the gene located at the left limit of the hemizygous region in A. sinica and A. urmiana was analysed separately. In A, the observed SNPs topologies. In B, the ten exons are illustrated in alterning black and gray segments. The first 4 exons correspond to a mediator of RNA polymerase II transcription subunit 27-like and the last 5 exons to a small ribosomal subunit protein uS7-like. This two genes were likely wrongly annotated in the genome annotation that we used. Those two genes show very different topologies with the one one the right clearly showing several SNPs with per chromosome topologies. The limit of S0 is therefore probably between those two genes.*

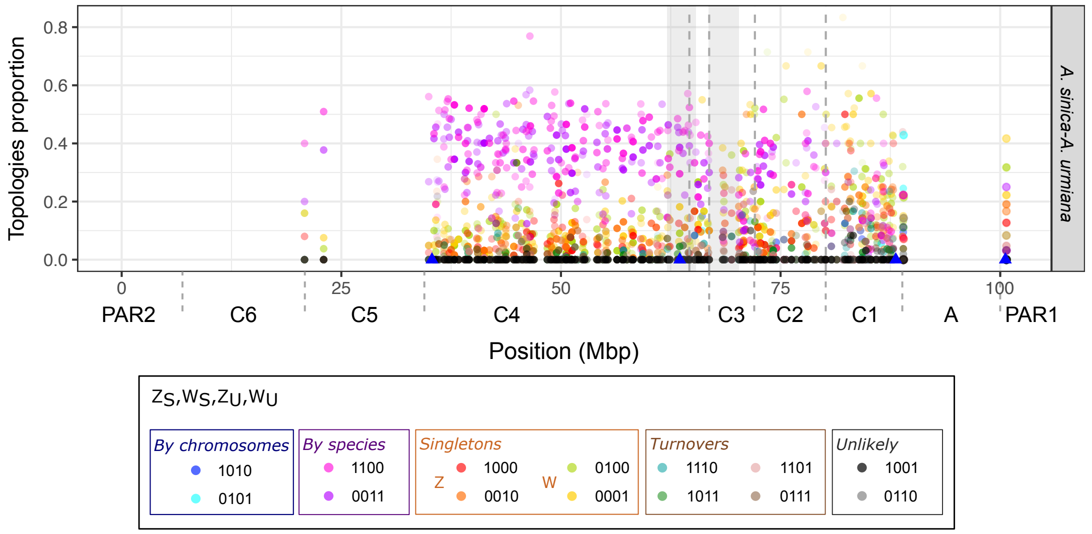

Figure S10 Raw topologies for *Artemia* species pair. *Same as Fig. 5, bottom panel, but showing results including the outliers and with raw topologies per gene instead of per region averaging.*

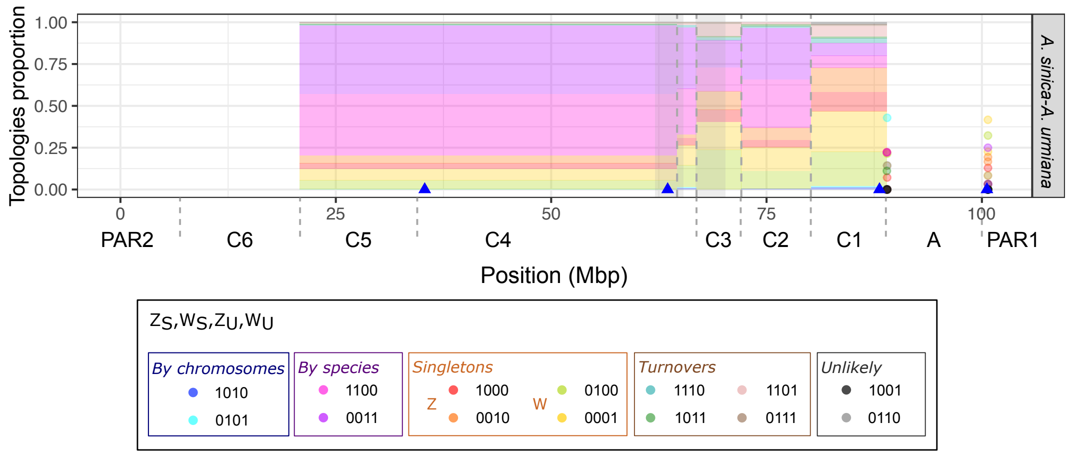

Figure S11 Topology change-point analysis for *Artemia* species. *Same as Fig. 5, bottom panel, but showing results including the outliers.*

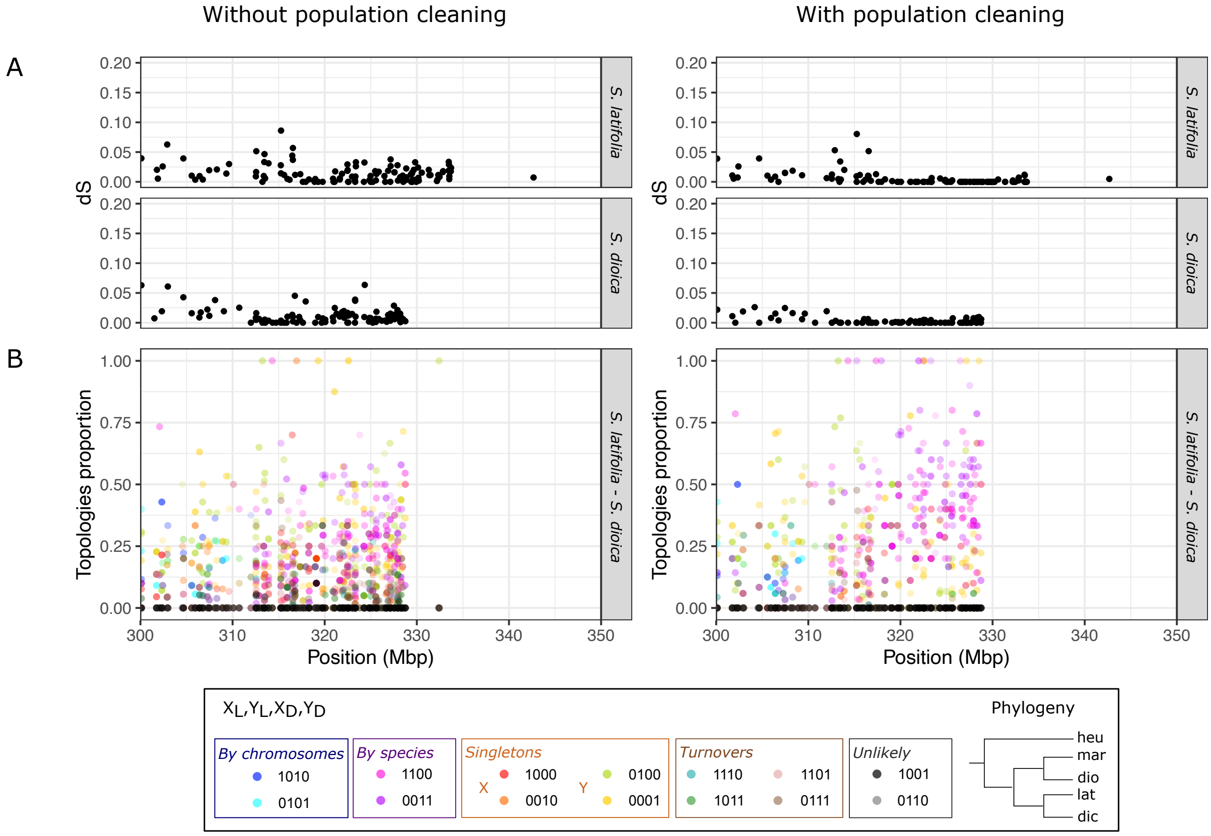

Figure S12 Population cleaning. *The figure illustrates the effect of removing false Y SNP using population data in S. latifolia and S. dioica. This cleaning procedure was the most efficient for those two species for which we had a relative large population sample (see text). The data without cleaning are represented on the left and the data with cleaning on the right. Panel A shows the dS (same code as in Fig. 4) and panel B shows the topologies (same code as in Fig. 2).*

Table S1. Read trimming. *The table shows how many bases were trimmed at the 5' and 3' ends of forward and reverse reads for all the data used in this paper. It also indicates whether additional steps, such as removing contamination or ribosomal RNA, were necessary.*

			1st Trimming						2nd Trimming						
			5'			3'			5'			3'			Additional step
	Species name	Forward/ Reverse	run1	run2	run3	run1	run2	run3	run1	run2	run3	run1	run2	run3	
Crosses	*S. diclinis*	Forward	12	15	12	0	0	0	0	0	0	0	2	2	
		Reverse	12	15	12	20	50	30	0	0	0	0	0	4	
	S. dioica	Forward	12	12	-	4	4	-	0	0	-	0	0	-	
		Reverse	12	12	-	4	4	-	0	0	-	0	0	-	
	S. heuffelii	Forward	11	11	12	4	0	4	0	2	5	4	0	0	
		Reverse	11	11	12	4	50	4	0	2	0	4	0	0	
	S. latifolia	Forward	13	-	-	0	-	-	0	-	-	4	-	-	no second trimming for the mother U37
		Reverse	13	-	-	0	-	-	0	-	-	4	-	-	
	S. marizii	Forward	12	12	10	0	4	4	0	0	0	0	0	0	
		Reverse	12	12	10	0	50	15	0	0	0	0	0	0	
	A. sinica ∗	Forward	10	10	-	10	10	-	0	0	0	0	0	0	
		Reverse	10	10	-	10	10	-	0	0	0	0	0	0	
	A. urmiana ∗	Forward	10	10	-	10	10	-	0	0	0	0	0	0	removal of human contamination for M5_urm1230_male
		Reverse	10	10	-	10	10	-	0	0	0	0	0	0	
Population	*S. diclinis*	Forward	15	-	-	0	-	-	0	-	-	2	-	-	
		Reverse	15	-	-	0	-	-	0	-	-	2	-	-	
	S. dioica	Forward	15	-	-	2	-	-	0	-	-	0	-	-	for female NORA, 24 cutted in 5' and removal of rRNA for SD_VS1_8 and SD_VS1_5
		Reverse	15	-	-	2	-	-	0	-	-	0	-		
	S. heuffelii	Forward	15	-	-	2	-	-	0	-	-	0	-	-	
		Reverse	15	-	-	2	-	-	0	-	-	0	-	-	
	S. latifolia pop1 ∗∗	Forward	12	-	-	3	-	-	0	-	-	0	-	-	
		Reverse	12	-	-	3	-	-	0	-	-	0	-	-	
	S. latifolia pop2 ∗ ∗ ∗	Forward	15	-	-	2	-	-	0	-	-	0	-	-	
		Reverse	15	-	-	2	-	-	0	-	-	0	-	-	
	S. marizii	Forward	12	-	-	2	-	-	0	-	-	0	-	-	
		Reverse	12	-	-	2	-	-	0	-	-	0	-	-	
Outgroup	*S. viscosa*	Forward	13	-	-	0	-	-	0	-	-	0	-	-	
		Reverse	13	-	-	0	-	-	0	-	-	0	-	-	
	A. franciscana	Forward	12	-	-	2	-	-	0	-	-	0	-	-	
		Reverse	12	-	-	2	-	-	0	-	-	0	-	-	

∗trimming done using `Cutadapt` instead of `Trimgalore`

∗∗Group of three females from Switzerland : *SL_GR2_2_female*, *SL_VS2__female*, *SL_TI1_2_female*

∗ ∗ ∗Group of females from whole Europa : *SL_AUST*, *SL_BUL*, *SL_FRA_Orsay*, *SL_FRA_Pyr*, *SL_IT_1*, *SL_IT_2*, *SL_POL*, *SL_PORT*, *SL_RUM*, *SL_RUS_F*, *SL_SPAN*, *SL_SWI_Engadin*, *SL_SWI_Leuk*, *SL_SWI_Verzasca*

Table S2. Topology proportions per region. *The table reports the topology proportions per region delimited by the change-point analysis for each species pair studied in Figs. 2 and 5. The table lists the pair of species studied, the block number (from left to right in the figures), the left and right limits of each block, and the proportion of each of the 14 topologies, coded as in Fig. 1. Line 1, 2 and last of the file are indicated below. For the full table, see supplementary file* `table_S2.xlsx`

pair	bloc	left limit	right limit	1010	0101	0100	0001	1000	0010	1100	0011	1110	1101	1011	0111	1001	0110
sin_urm	1	20816495	66872523	0	0	0.06	0.07	0.03	0.04	0.37	0.42	0.01	0	0	0	0	0
...																	
heu_mar	12	312570020	338397814	0	0	0.17	0.09	0.01	0.02	0.25	0.41	0.01	0.01	0.02	0.01	0	0

Table S3. Summary of region limits in *Artemia*. *The table indicates the position (bp) of the limits of S0, C1 to C6 regions. The limits between C1 to C4 regions are provided by the change-point analysis on topologies. The limits between C4 to C6 and the PAR2 correspond to the position of last sex-linked gene observed (see Fig. 5). The limit between S0 and C1 is within the G gene (see Fig. S9).*

Regions limit	Position (bp)
PAR1-S0	100700310
S0-C1	$[88986000 - 88997192]$
C1-C2	$80147748[80110002 - 80183005]$
C2-C3	$72015637[72015637 - 72015637]$
C3-C4	$66872523[66792152 - 66872523]$
C4-C5	34950767
C5-C6	20367848
C6-PAR2	7290006

Table S4. Summary of region limits in *Silene*. *The table indicates the position (bp) of the limits of A1, P, A2, A3, L regions. Those limits are not exactly the same when analysing each species pair separately. For each limit, we report the average across all species. We also report the minimum and maximum position observed among species pairs together with their support limits.*

Regions limit	Position (bp)		
	average	minimum	maximum
A1-P	7241148	$6797838[6797838 - 6805728]$	$7510565[7379247 - 7510565]$
P-A1	10165991	$9976636[9976636 - 9976636]$	$10602363[10602363 - 10602363]$
A1-A2	23739065	$22872189[21951730 - 24377344]$	$24391147[24391147 - 24391147]$
A2-A3	279228730	$274953092[274953092 - 276672441]$	$283410506[281928757 - 286113316]$
A3-L	311466571	$309320796[309320796 - 310722267]$	$312578174[312561594 - 312871196]$
L-PAR	316667303	$316543069[316543069 - 316543069]$	$316789380[316758115 - 316789380]$
